# FUS regulates AMPA receptor function and FTLD/ALS-associated behaviour via GluA1 mRNA stabilization

**DOI:** 10.1038/ncomms8098

**Published:** 2015-05-13

**Authors:** Tsuyoshi Udagawa, Yusuke Fujioka, Motoki Tanaka, Daiyu Honda, Satoshi Yokoi, Yuichi Riku, Daisuke Ibi, Taku Nagai, Kiyofumi Yamada, Hirohisa Watanabe, Masahisa Katsuno, Toshifumi Inada, Kinji Ohno, Masahiro Sokabe, Haruo Okado, Shinsuke Ishigaki, Gen Sobue

**Affiliations:** 1 Department of Neurology, Nagoya University Graduate School of Medicine, Nagoya 466-8550, Japan; 2 Graduate School of pharmaceutical Sciences, Tohoku University, Sendai 980-8578, Japan; 3 Mechanobiology Laboratory, Nagoya University Graduate School of Medicine, Nagoya 466-8550, Japan; 4 Department of Neuropsychopharmacology and Hospital Pharmacy, Nagoya University Graduate School of Medicine, Nagoya 466-8550, Japan; 5 Division of Neurogenetics, Center for Neurological Diseases and Cancer, Nagoya University Graduate School of Medicine, Nagoya 466-8550, Japan; 6 Department of Brain Development and Neural Regeneration, Tokyo Metropolitan Institute of Medical Science, Tokyo 156-8506, Japan

## Abstract

FUS is an RNA/DNA-binding protein involved in multiple steps of gene expression and is associated with amyotrophic lateral sclerosis (ALS) and fronto-temporal lobar degeneration (FTLD). However, the specific disease-causing and/or modifying mechanism mediated by FUS is largely unknown. Here we evaluate intrinsic roles of FUS on synaptic functions and animal behaviours. We find that FUS depletion downregulates GluA1, a subunit of AMPA receptor. FUS binds GluA1 mRNA in the vicinity of the 3′ terminus and controls poly (A) tail maintenance, thus regulating stability. GluA1 reduction upon FUS knockdown reduces miniature EPSC amplitude both in cultured neurons and *in vivo*. FUS knockdown in hippocampus attenuates dendritic spine maturation and causes behavioural aberrations including hyperactivity, disinhibition and social interaction defects, which are partly ameliorated by GluA1 reintroduction. These results highlight the pivotal role of FUS in regulating GluA1 mRNA stability, post-synaptic function and FTLD-like animal behaviours.

## Abstract (web summary)

FUS is an RNA/DNA-binding protein involved in gene expression regulation and associated with amyotrophic lateral sclerosis and frontotemporal dementia (FTLD) but the disease-causing mechanisms are unclear. Here the authors show that FUS regulates the stability of *GluA1* mRNA and dendritic maturation and plays a role in FTLD-associated behaviours.

## Graphical abstract

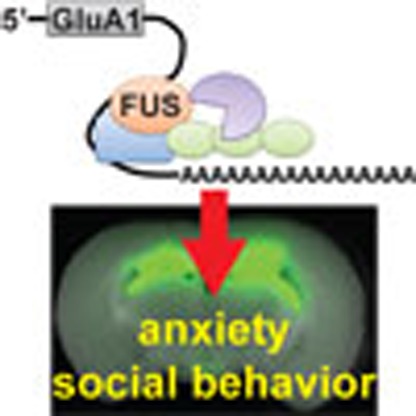

FUS, originally identified as a fusion protein caused by a chromosomal translocation in human liposarcomas^1^, is an RNA/DNA-binding protein implicated in multiple steps of gene expression. FUS was later independently isolated as a gene mutated in familial amyotrophic lateral sclerosis (ALS). FUS-containing protein aggregates were found in the patients of both familial and sporadic cases of ALS and frontotemporal dementia (FTLD)^2^,^3^,^4^,^5^. ALS and FTLD are two different neurodegenerative diseases. However, it is becoming obvious that these diseases are on the same spectrum of disorders, sharing the same disease-causing factors including FUS and another RNA-binding protein TDP-43 (ref. 6). A number of studies have suggested that aberrant RNA metabolism caused by the loss of these RNA-binding proteins inhibits normal neuronal functions and affects disease symptoms^6^,^7^.

Several groups have identified FUS target mRNAs by high-throughput sequencing of RNA isolated by crosslinking immunoprecipitation (HITS-CLIP)^8^,^9^,^10^. These reports demonstrated that FUS binds long introns and near exon–intron junctions, suggesting a role in splicing. According to the HITS-CLIP data, a significant population of FUS also interacts with mRNA 3′ untranslated regions (UTRs)^9^. FUS is present both in the soma and the neuronal processes and was shown to affect the transport of certain mRNAs along dendrites^11^ and translation in neuronal protrusions^12^. These studies have suggested that FUS binds to and regulates multitude of mRNAs in neurons, however, the mechanism by which it regulates these post-transcriptional events is largely unknown and little is known about specific targets that are critical for the disease phenotypes^7^.

In this study, we evaluate intrinsic roles of FUS on synaptic functions and animal behaviours and aim to identify the specific factor regulated by FUS that is crucial for those functions. First, we examined the expression pattern of synaptic proteins and found that FUS depletion specifically downregulates AMPA (α-amino-3-hydroxy-5-methyl-4-isoxazole propionic acid) receptor subunit, GluA1. FUS binds a region near the 3′ end of GluA1 mRNA and regulates its stability via the control of poly (A) tail length (PAT). We further show that FUS modulates synaptic transmission both in cultures and *in vivo*. FUS knockdown in the hippocampus causes a deficit in synapse maturation and behavioural aberrations, which are reminiscent of FTLD symptoms and are partly ameliorated by exogenous GluA1. The confluence of these and other findings indicates that the FUS-containing 3′-end processing machinery regulates GluA1 mRNA stability, which we suggest represents a coherent molecular mechanism that underlies synapse morphogenesis and FTLD-related animal behaviours.

## Results

### FUS knockdown downregulates GluA1 expression

**Figure 1:**
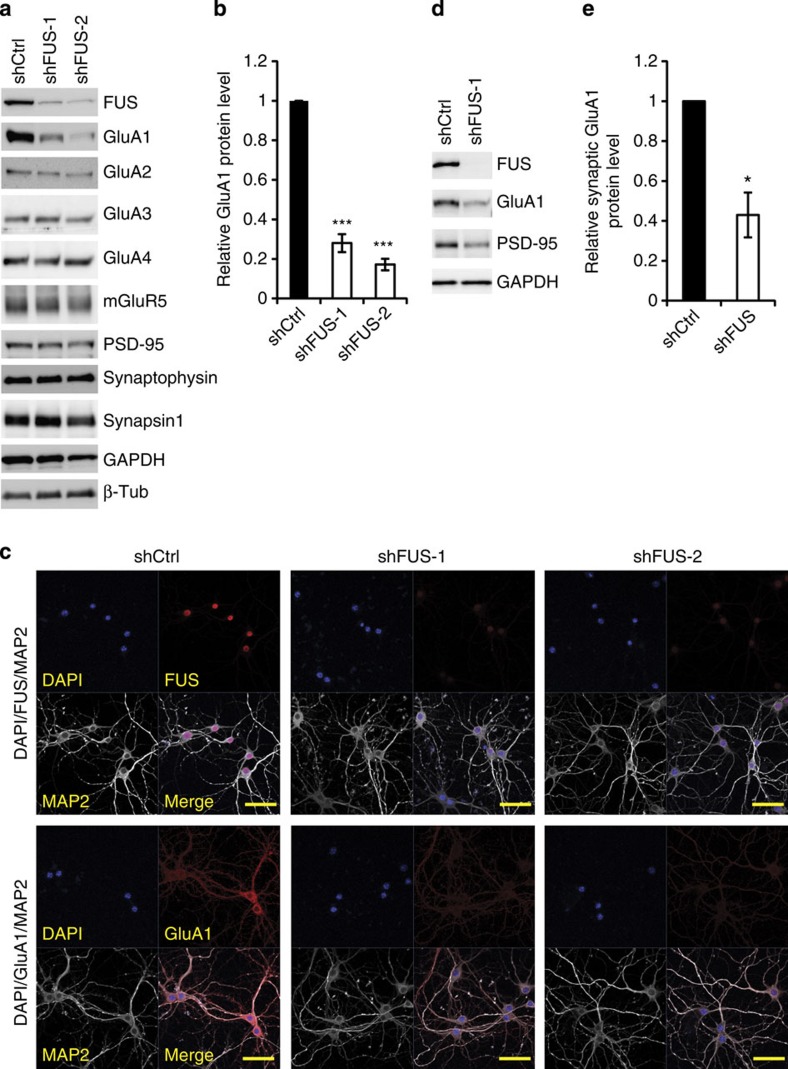
FUS depletion downregulates GluA1 protein level in cultured neurons. (**a**) Mouse cultured cortical neurons were infected by lentiviruses expressing scrambled shRNA (shCtrl) or shRNA for FUS (shFUS-1 and shFUS-2) at DIV 10. Ten days post infection, the lysates were prepared and protein levels were analysed by western blot using the antibodies indicated. (**b**) Quantification of GluA1 protein levels in **a** (*n*=4; F(2, 9)=298.4, *P*<0.0001, one-way analysis of variance; ***P<0.001, Tukey's test *post hoc*). (**c**) Primary hippocampal neurons were transduced as in **a** and immunostained for MAP2 and FUS or GluA1 together with 4,6-diamidino-2-phenylindole (DAPI) staining. Scale bar, 50 μm. (**d**) Synaptoneurosomes were prepared from the control (shCtrl) and FUS knockdown (shFUS-1) primary cortical neurons and western blotted with the indicated antibodies. (**e**) Quantification of synaptic GluA1 protein levels in **d** (*n*=3, *t*=5.063 **P=0.037, paired *t*-test).

To elucidate the impact of FUS loss on synaptic functions, we depleted endogenous FUS in primary neurons by short hairpin RNA (shRNA)-expressing lentiviruses. Lentiviruses were infected at day 10 *in vitro* and the lysates were prepared 10 days post infection. Among the proteins examined, including pre- and post-synaptic proteins (Synapsin, Synaptophysin, GluA1, GluA2, GluA3, GluA4, Grin2A, Grin2B, mGluR5 and PSD-95) and house-keeping factors (GAPDH and β-Tubulin), we found that the AMPA receptor subunit, GluA1, was significantly downregulated upon FUS knockdown in primary cortical and hippocampal neurons (Fig. 1a, b and Supplementary Fig. 1a–c). Second shRNA against FUS reduced GluA1 level to a similar extent, excluding the possible effect of off-targeting. Immunostaining showed an overall reduction in GluA1 signal throughout the cell (Fig. 1c). Long (15–19 days) and efficient FUS knockdown marginally reduced other post-synaptic proteins GluA2 and Grin2A (Supplementary Fig. 1c). These are likely secondary effects of GluA1 reduction because GluA1 knockdown by shRNA also reduced the levels of these proteins (Supplementary Fig. 1d). We also examined the GluA1 protein level in synaptoneurosomes and confirmed that synaptic GluA1 was reduced by FUS depletion (Fig. 1d,e).

### FUS regulates GluA1 mRNA stability

**Figure 2:**
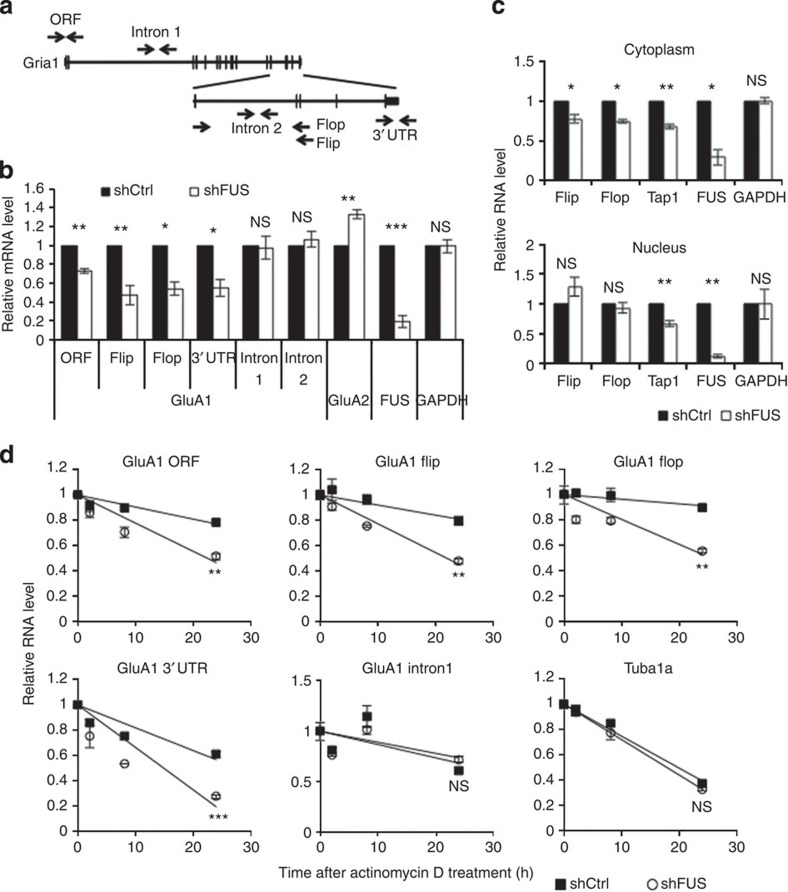
GluA1 mRNA stability is reduced upon FUS depletion. (**a**) Location of GluA1 qPCR primers used for quantification of different forms of GluA1 transcripts. Primer pairs for intron 1 and intron 2 detect only primary (unspliced) GluA1 transcripts, whereas primer pairs, ORF, flop, flip and 3′UTR detect spliced mature mRNAs. (**b**) Cultured cortical neurons were transduced by lentiviruses expressing shCtrl or shFUS as described in Fig. 1a. Total RNA was extracted and used for qRT–PCR using the primers described in **a** and the primers for FUS and GAPDH (ORF: *n*=3, *t*=10.12, **P=0.0096; flip: *n*=4, *t*=5.902, **P=0.0097; flop: *n*=3, *t*=6.391, **P=0.024; 3′UTR: *n*=3, *t*=4.985, *P=0.038; intron1: *n*=4, *t*=0.2265, *P*=0.8353; intron 2: *n*=3, *t*=0.7341, *P*=0.54; FUS: *n*=3, *t*=12.75, **P=0.0061; GAPDH: *n*=4, *t*=0.1446, *P*=0.89, paired *t*-test). (**c**) Cytoplasmic and nuclear RNA were separately prepared from control and FUS knockdown cortical neurons and RNA from each fraction was subjected for qRT–PCR as in **b** (cytoplasm, flip: *t*=5.856, *P=0.028; flop: *t*=5.966, *P=0.027; Tap1: *t*=17.81, **P=0.0031; FUS: *t*=9.368, *P=0.011; GAPDH: *t*=1.027, *P*=0.4124; nucleus, flip: *t*=1.831, *P*=0.21; flop: *t*=0.8251, *P*=0.50; Tap1: *t*=6.529, **P=0.023; FUS: *t*=26.23, **P=0.0014; GAPDH: *t*=1.818, *P*=0.2107, *n*=3, paired *t*-test). (**d**) mRNA stability was measured in the control and FUS knockdown primary cortical neurons treated with 10 μg ml^−1^ actinomycin D by qRT–PCR using primer sets indicated above each graph. The RNA level relative to pretreatment in equal amount of total RNA was plotted against time after the treatment. Residual RNA levels at 24 h were compared (*n*=3 each, GluA1 ORF: *t*=8.184, **P=0.0012; GluA1 flip: *t*=5.500, **P=0.0053; GluA1 flop: *t*=7.365, **P=0.0018; GluA1 3′UTR: *t*=10.41, ***P=0.0005; GluA1 intron1: *t*=1.527, *P*=0.20; Tuba1a: *t*=2.654, *P*=0.057, unpaired *t*-test). GAPDH, glyceraldehyde 3-phosphate dehydrogenase; NS, not significant.

Next, we examined at which step FUS regulates GluA1 expression as FUS is implicated in multiple steps of gene regulation. GluA1 transcript level was examined by reverse transcription followed by quantitative reverse transcription–PCR (qRT–PCR) using four primer sets targeting flip and flop exons, which detect alternative splicing variants, and the third exon open reading frame (ORF) and 3′ UTR, which detect total GluA1 mRNA (Fig. 2a). The quantitative PCR (qPCR) with these primers showed that FUS depletion reduced GluA1 mature mRNA level (Fig. 2b). We then used two additional primer sets against intron sequences to detect GluA1 primary transcripts (Fig. 2a). qPCR for intron sequences did not reduce GluA1 RNA level, suggesting that GluA1 transcription was not affected (Fig. 2b). Second shRNA against FUS had a similar effect on GluA1 transcript levels (Supplementary Fig. 2). Moreover, we extracted cytoplasmic and nuclear RNAs and measured GluA1 transcript levels in each fraction (Fig. 2c). GluA1 mature transcripts were reduced by FUS depletion only in the cytoplasm, whereas Tap1 mRNA, previously shown to be downregulated by FUS knockdown, was reduced both in the cytoplasm and nucleus. These data indicate that FUS depletion post-transcriptionally downregulates GluA1 in the cytoplasm. A previous report showed that FUS also regulates GluA1 mRNA flip and flop alternative splicing; upon FUS depletion the flip isoform increases^13^. We also observed a similar trend only in the nucleus, although the change was short of statistical significance (Fig. 2c).

We then measured GluA1 mRNA stability by qRT–PCR on control and FUS knockdown neurons treated with the transcriptional inhibitor actinomycin D or α-amanitin (Fig. 2d and Supplementary Fig. 3). GluA1 mature mRNA was significantly more destabilized by FUS knockdown, whereas stability of GluA1 primary transcript, other mRNAs encoding synaptic proteins (Grin2A, Grin2B and Synaptophysin) and control Tubulin (Tuba1a) mRNA, was unaffected. A previous report demonstrated that FUS facilitates the transport of certain mRNAs in dendrites^11^. Therefore, we examined whether the transport of GluA1 mRNA was also regulated by FUS by fluorescent *in situ* hybridization (FISH; Supplementary Fig. 4). GluA1 FISH signal was quantified in the soma and distal dendrites 30–100 μm away from the soma. FUS knockdown decreased the GluA1 FISH signal in the soma, but not in the dendrites, suggesting that GluA1 mRNA transport was not interrupted by FUS depletion. These results together indicate that FUS regulates GluA1 expression by stabilizing GluA1 mature mRNA in the cytoplasm.

### FUS binds GluA1 mRNA in the vicinity of the 3′ terminus

**Figure 3:**
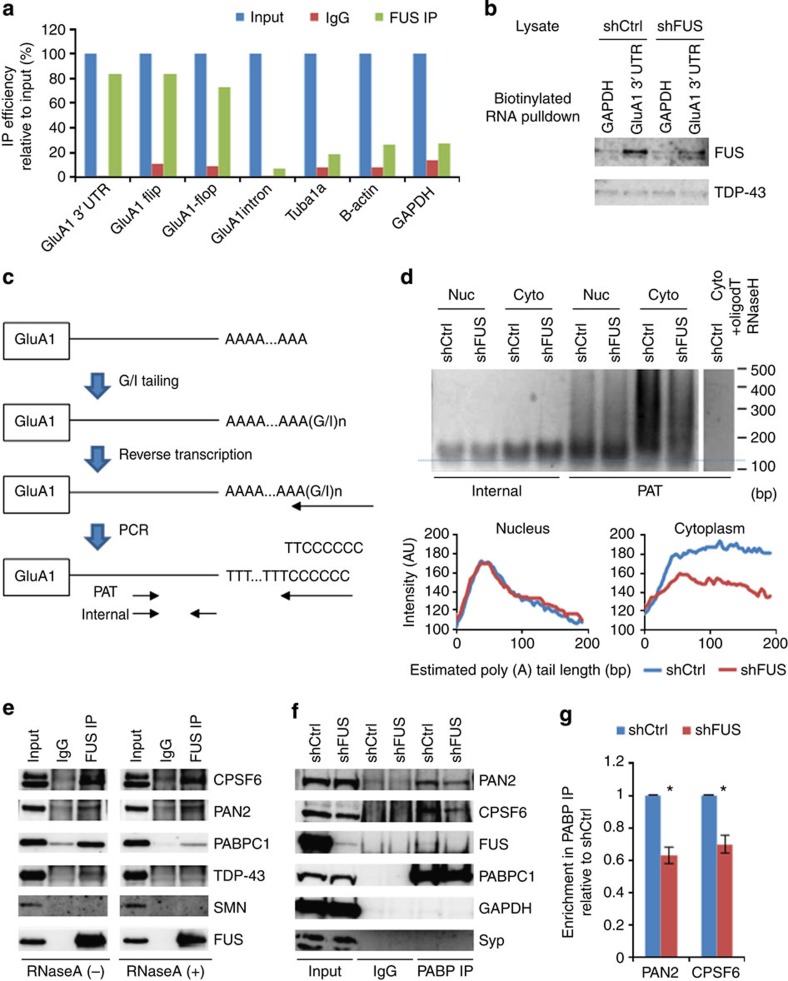
FUS-containing 3′ UTR processing machinery modulates GluA1 mRNA polyadenylation. (**a**) RNA IP of FUS in cultured cortical neurons. Bound RNA was analysed by RT–PCR using the indicated primers. IP efficiency was calculated relative to input. Representative data from triplicate experiments are shown. (**b**) Biotinylated RNA pull-down assay to assess endogenous FUS binding to biotinylated RNA for GluA1 3′UTR or control GAPDH mRNA. Each biotinylated RNA was incubated with lysate either from control or from FUS knockdown cortical neurons and analysed by western blot. TDP-43 served as a negative control. (**c**) Scheme of GluA1 mRNA poly (A) tail length (PAT) analysis. (**d**) The polyadenylation assay was performed using nuclear (Nuc) and cytoplasmic (Cyto) RNA from control and FUS knockdown cortical neurons. Cytoplasmic RNA from control cortical neurons was treated with RNase H in the presence of oligo dT and served as negative control. Smear signals were quantified by ImageJ software and plotted against the length of poly (A) tail calculated based on the size marker. (**e**) Co-IP of FUS in mouse brain lysate with or without RNase A treatment. Bound proteins were detected by western blot using the indicated antibodies. (**f**) Co-IP of PABPC1 using lyates from FUS knockdown and control primary cortical neurons. (**g**) Quantification of PAN2 and CPSF6 Co-IP levels in **f** (*n*=3; PAN2: *t*=7.341, *P=0.018; CPSF6: *t*=5.556, *P=0.031, paired *t*-test). GAPDH, glyceraldehyde 3-phosphate dehydrogenase.

The data above revealed a novel function of FUS in controlling GluA1 mRNA stability. To elucidate the mechanism by which FUS regulates GluA1 mRNA stability, we established whether FUS binds GluA1 mRNA (Fig. 3a). Immunoprecipitation (IP) of FUS from primary neurons followed by RNA extraction and reverse transcription–PCR (RT–PCR) demonstrated that FUS binds GluA1 mature mRNA; both flip and flop transcripts were enriched in the pull-down fraction. GluA1 primary transcript was not efficiently pulled down with FUS, further confirming that FUS regulates GluA1 mature mRNA stability.

As mRNA stability is often regulated at the 3′ UTR, we examined the reported FUS CLIP tag sequences on the GluA1 3′ UTR^9^ (Supplementary Fig. 5). There was a significant peak of CLIP tag enrichment near the 3′ end of the genome-coded mRNA sequence. The ∼350 bases of sequence spanning this area of enrichment was highly conserved among mammals. A streptavidin pull-down assay with *in vitro* transcribed biotinylated RNA corresponding to this 350 bases of sequence was performed using primary neuron lysates, and indeed this region bound to endogenous FUS (Fig. 3b). Moreover, a reporter assay using mRNA-encoding Venus fluorescence protein fused with this GluA1 3′ UTR sequence displayed a reduction of reporter expression upon FUS knockdown (Supplementary Fig. 6). Together, these data indicate that FUS binds GluA1 mRNA near the 3′ terminus and regulates its stability.

### FUS regulates GluA1 mRNA polyadenylation

One possible mechanism by which FUS regulates GluA1 mRNA stability is the control of PAT as it binds GluA1 mRNA near the 3′ terminus. Therefore, we measured PAT of GluA1 mRNA by PCR-based PAT assay (Fig. 3c). The poly (A) tail is detected as a smear above the band corresponding to size of the internal control (Fig. 3d). In the nuclear fraction, there was no difference in the pattern and intensity of the smears. However, in the cytoplasm, the smear intensity was weaker and shifted towards shorter poly (A) tails in the FUS knockdown. Incubation of the RNA with oligo (dT) followed by RNase H digestion diminished the smear, proving that the smear represents the poly (A) tails. Note that the band intensity for internal PCR was indistinguishable between the control and FUS knockdown, indicating that the PCR cycle was saturated and therefore the reduced poly (A) smear intensity in the FUS knockdown is not due to the reduced transcript level. Poly (A) tail of GluA1 mRNA was also examined by poly (A) RNA fractionation using biotinylated oligo (dT) followed by elutions with the buffers with a different salt concentration and qRT–PCR^14^. Indeed, GluA1 mRNA with long poly (A) tail was significantly reduced upon FUS depletion (Supplementary Fig. 7). These data strongly suggest that FUS regulates GluA1 mRNA stability through the maintenance of PAT.

Because FUS has no known activity related to poly (A) tail lengthening, it likely forms a complex with the factors involved in 3′-end processing and/or polyadenylation. We performed IP of FUS in neuroblastoma cells and identified associated proteins by mass-spectrometry (Supplementary Table 1). Identified proteins included splicing factors. In addition, there were many cytoplasmic RNA-binding proteins and factors involved in mRNA 3′-end processing, polyadenylation and translation. Among them, we confirmed that cleavage and polyadenylation-specific factor CPSF6, poly (A)-specific ribonuclease PAN2 and cytoplasmic poly (A)-binding protein PABPC1 (ref. 15) were associated with FUS in the brain (Fig. 3e), primary neurons and neuroblastoma cells (Supplementary Fig. 8). Moreover, CPSF6 and PAN2 interacted with FUS both in the presence and absence of RNase A, indicating that they form an RNA-independent protein complex (Fig. 3e). We examined the interaction of these factors separately in the cytoplasm and the nucleus of cultured hippocampal neurons (Supplementary Fig 8e). Although PABPC1 and CPSF6 are reportedly localized in the cytoplasm and the nucleus, respectively, substantial amount of these proteins were present in both fractions. FUS Co-IPed with CPSF6 and PABPC1 both in the cytoplasm and the nucleus, whereas PAN2 was predominantly bound by FUS in the cytoplasm.

We then examined if FUS depletion affects the integrity of this FUS-containing protein complex. We found that PABPC1 co-IPed with PAN2 and CPSF6 and these interactions were diminished upon FUS depletion (Fig. 3f,g and Supplementary Fig. 8c). These data suggest that FUS sequesters PAN2 on PABPC1 and prevents it from deadenylating the poly (A) tail of the bound mRNA (Supplementary Fig. 9). Indeed, the knockdown of PAN2 increased the GluA1 protein and mature mRNA levels, while leaving the primary transcript level unchanged (Supplementary Fig. 10). Thus, these data strongly suggest that the FUS-containing 3′-end processing machinery regulates GluA1 mRNA stability via the control of PAT.

### FUS depletion in cultures affects basal synaptic transmission

**Figure 4:**
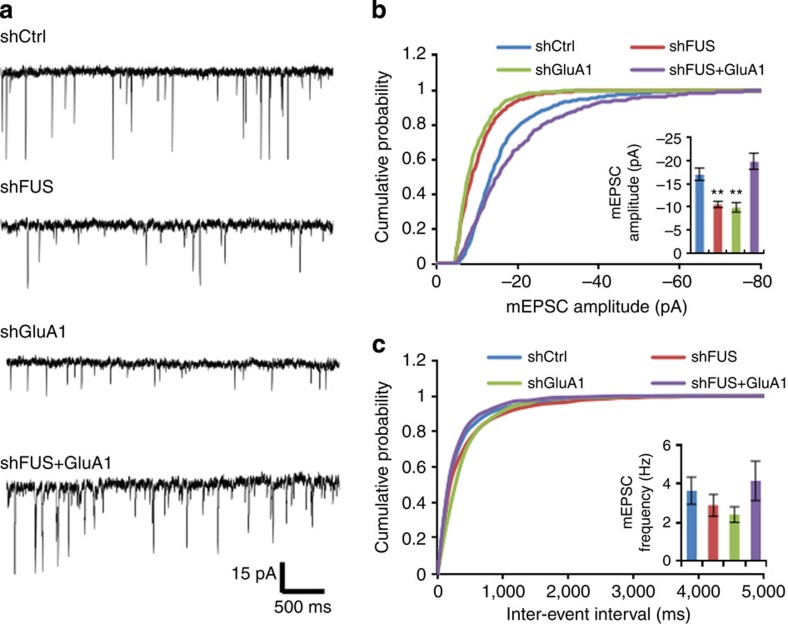
FUS knockdown neurons exhibit reduced AMPA receptor surface expression and mEPSC amplitude. (**a**) Representative mEPSC sample traces from the control (*n*=9), FUS knockdown (*n*=8), GluA1 knockdown (*n*=4) and FUS knockdown with GluA1 expression (*n*=5) cultured cortical neurons. One to three cells per separate culture were used for experiments. Calibration: 15 pA, 500 ms. (**b**, **c**) Cumulative distributions of mEPSC amplitude (**b**) and frequency (**c**) from the control, FUS knockdown, GluA1 knockdown and FUS knockdown with GluA1 expression cultured cortical neurons. The insets show mean±s.e.m. for mEPSC amplitude (F(3, 22)=12.50, *P*<0.0001, one-way analysis of variance (ANOVA); shCtrl versus shFUS: **P=0.0028, shCtrl versus shGluR1: **P=0.0084, Tukey's test *post hoc*; **b**) and frequency (F(3, 22)=0.8617, *P*=0.4756, one-way ANOVA; **c**).

The data presented thus far indicate that FUS regulates GluA1 mRNA stability and protein expression. As synaptic AMPA receptor expression is critical for synaptic transmission and maturation^16^,^17^, next we directly measured miniature excitatory post-synaptic current (mEPSC) in control and FUS knockdown primary neurons (Fig. 4a–c). mEPSC amplitude is indicative of the strength of mature synapses, whereas mEPSC frequency is dependent on synapse number and presynaptic release probability. We found that FUS depletion significantly reduced mEPSC amplitude, while keeping the frequency intact. Second shRNA against FUS had a similar effect (Supplementary Fig. 11). Moreover, GluA1 knockdown was indistinguishable to the FUS depletion and concomitant expression of GluA1 in the FUS knockdown (Supplementary Fig. 12) recovered mEPSC amplitude (Fig. 4b), demonstrating that FUS-mediated GluA1 expression is indeed critical for synaptic transmission.

Unexpectedly, however, forskolin/roliplam-induced long-term potentiation in cultured hippocampal neurons induced a similar level of GluA1 serine 845 phosphorylation, whereas serine 831 phosphorylation level was reduced as much as total GluA1 level (Supplementary Fig. 13a). Likewise NMDA stimulation, which induces long-term depression, reduced serine 845 phosphorylation both in the control and the FUS knockdown (Supplementary Fig. 13b). These data suggest that the activity-dependent synaptic response in the FUS knockdown remains intact at least for serine 845 phosphorylation and that FUS knockdown and/or total GluA1 reduction might also affect GluA1 trafficking by an unknown mechanism.

### FUS depletion *in vivo* alters synaptic transmission and spine morphogenesis

The nexus of these observations indicates FUS plays pivotal roles in synaptic functions through the regulation of GluA1 mRNA stability in cultures. We next evaluated whether FUS depletion *in vivo* causes deficits of synaptic functions. To this end, we stereotactically injected adeno-associated viruses (AAVs) expressing shFUS into the mouse hippocampus, because the hippocampus is one of the most degenerated brain regions in FTLD/ALS patients^18^ and it is involved in multiple aspects of behaviour including learning and memory, emotion, anxiety, hyperactivity and social interaction^19^,^20^.

**Figure 5:**
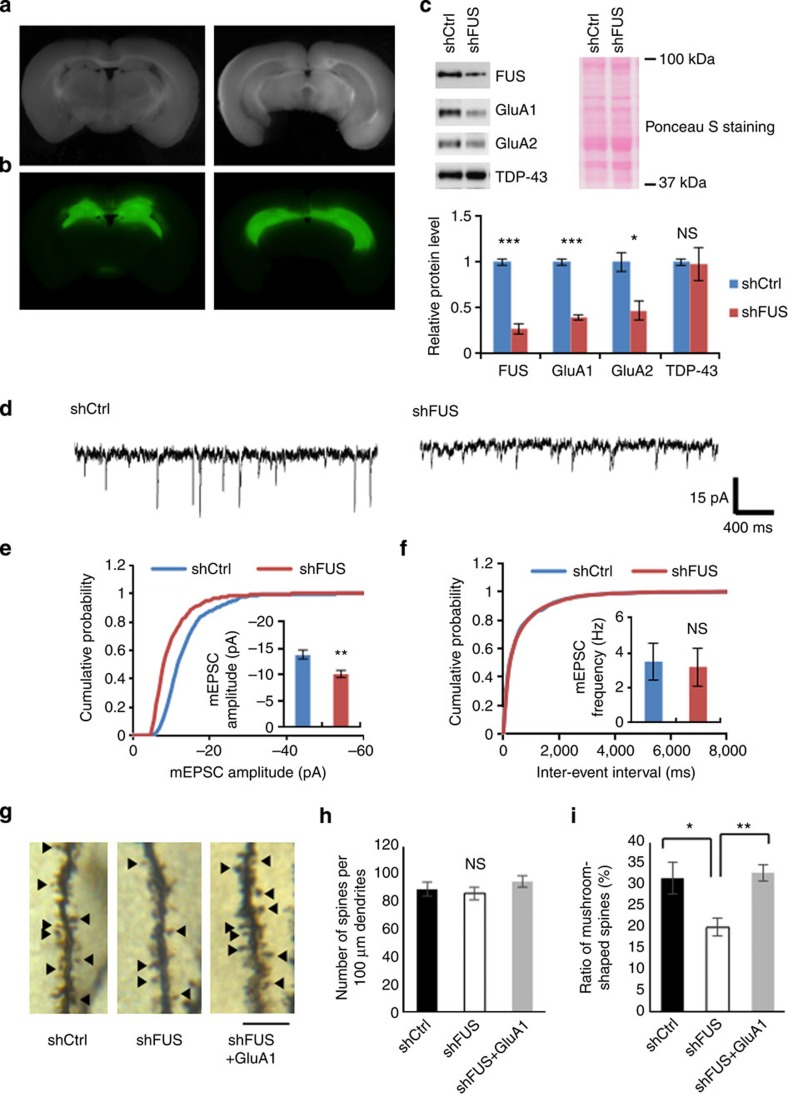
shRNA-mediated knockdown of FUS in mouse hippocampus affects synaptic transmission and spine morphogenesis at CA1 apical dendrites. (**a**, **b**) Representative images of AAV-injected brain sections photographed by bright-field (**a**) and fluorescence (**b**) microscopy. Images at anterior (left) and posterior (right) sections are representatively shown. (**c**) The lysates were prepared from two transduced ‘green' hippocampal slices per animal and western blotted with the indicated antibodies. Protein levels were quantified in three control and three FUS knockdown mice (FUS: *t*=10.40, ***P=0.0005; GluA1: *t*=13.24, ***P=0.0002; GluA2: *t*=3.593, *P=0.023; TDP-43: *t*=0.1447, *P*=0.89; unpaired *t*-test). Ponceau S staining indicates equal loading. (**d**) Representative mEPSC sample traces from the control (*n*=7) and FUS knockdown (*n*=7) hippocampal slices. (**e**, **f**) Cumulative distributions of mEPSC amplitude (**e**) and frequency (**f**) from the control and FUS knockdown hippocampal slices. The insets show mean±s.e.m. for mEPSC amplitude (*t*=3.400, **P=0.0053, unpaired *t*-test; **e**) and frequency (*t*=0.1946, *P*=0.85, unpaired *t*-test; **f**). (**g**) Representative images of a dendritic segment of CA1 pyramidal neurons of the hippocampus from mice injected with AAV-shCtrl (*n*=9 dendrites from three mice), AAV-shFUS (*n*=9 dendrites from three mice) and AAV-shFUS plus AAV-GluA1 (*n*=6 dendrites from three mice). Mushroom spines are indicated with arrowheads. Scale bar, 5 μm. (**h**) Spine number was quantified along a 100-μm segment from the origin of primary apical dendritic branches of Golgi-impregnated CA1 pyramidal neurons. No statistical difference was detected (*P*>0.05, one-way analysis of variance (ANOVA)). (**i**) Mushroom-shaped spines over the total spines as in **h** were counted (*F*(2, 21)=5.843, **P=0.0096, one-way ANOVA; *P<0.05, Tukey's test *post hoc*). NS, not significant.

As measured by the green fluorescent protein (GFP) expression from the AAV, the hippocampus was largely transduced by the injections with only occasional GFP expression in the surrounding brain regions (Fig. 5a,b). Western blot of the microdissected GFP-positive hippocampus demonstrated efficient knockdown of FUS and GluA1 reduction (Fig. 5c). GluA2 was also decreased likely due to the GluA1 reduction (see Supplementary Fig. 1b,c), whereas the levels of other synaptic proteins were not significantly affected (Supplementary Fig. 14).

We measured mEPSC of CA1 pyramidal neurons in the acute hippocampal slices prepared from the AAV-injected mice (Fig. 5d–f). Consistent with the results in cultured neurons, FUS depletion in the hippocampus reduced mEPSC amplitude, while leaving the frequency unchanged, indicating that FUS modulates synaptic transmission both in cultures and *in vivo*.

We then examined dendritic spine density and morphology at apical dendrites of CA1 pyramidal neurons by Golgi-Cox staining (Fig. 5g and Supplementary Fig. 15a). Spine number in the FUS knockdown was indistinguishable to that in the control (Fig. 5h and Supplementary Fig. 15b). The ratio of mushroom-shaped mature spines versus thin and stubby spines was significantly decreased by FUS depletion, indicating that FUS impacts dendritic spine maturation *in vivo* (Fig. 5i). Moreover, when GluA1-expressing AAV was co-injected with shFUS-expressing AAV (Supplementary Fig. 12), the ratio of mature spines was significantly increased, while spine density remained unchanged (Fig. 5h,i). These data indicate that FUS is required for the maintenance of mature dendritic spines *in vivo*, via the stabilization of GluA1 expression.

### Social interaction defects in the FUS knockdown mice

**Figure 6:**
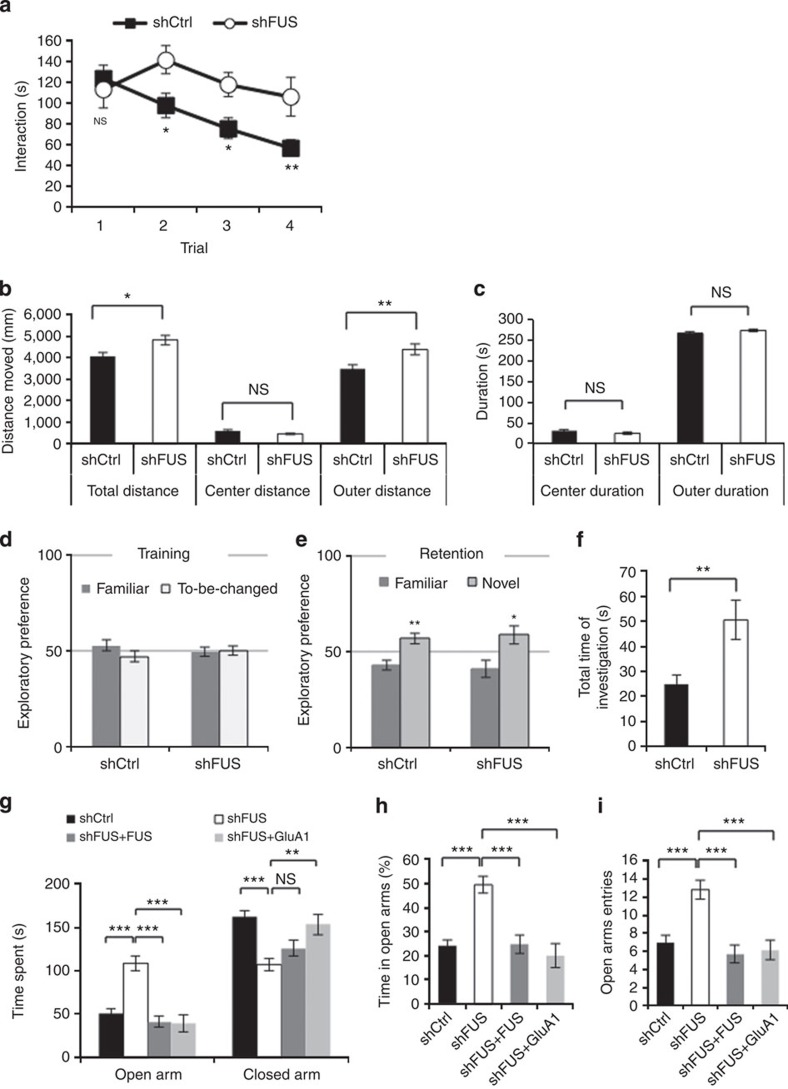
FUS knockdown in hippocampus induces behavioural aberrations related to FTLD symptoms. (**a**) Resident–intruder test measuring the investigation time of the test ‘resident' mouse of the AAV-shCtrl- (*n*=19) or AAV-shFUS-injected group (*n*=12) on an ‘intruder' wild-type mouse in four consecutive sessions (session 1: *t*=0.3070, *P*=0.76, session 2: *t*=2.739, *P=0.011, session 3: *t*=2.672, *P=0.76, session 4: *t*=2.929, **P=0.0067, unpaired *t* test). (**b**) Distance moved in the indicated compartment of an open field during the 5 min test session (*n*=11 each, total: *t*=2.601, *P=0.017; centre: *t*=1.841, *P*=0.081; outer: *t*=2.903, **P=0.0088; unpaired *t*-test). (**c**) Time spent in the centre and outer compartment of an open field. No statistical difference was detected (*P*>0.05, unpaired *t*-test). (**d**–**f**) Novel object recognition test. (**d**) In the training session, both shCtrl and shFUS groups (*n*=11 each) equally investigated two different objects placed in the test field. (*P*>0.05, unpaired *t*-test) (**e**) During the retention session where one object was replaced with a novel object, both shCtrl and shFUS mice spent significantly more time investigating the novel object (shCtrl: *t*=3.572, **P=0.0019; shFUS: *t*=2.663, *P=0.015, unpaired *t*-test). (**f**) Total investigation time on the two objects presented in training session (**P=0.0057, Mann–Whitney's *U*-test). (**g**–**i**) Elevated plus maze assay. (**g**) Time spent in the open and the closed arm of an elevated plus maze was measured for shCtrl (*n*=21), shFUS (*n*=23), shFUS plus shFUS-resistant FUS expression (*n*=12) and shFUS plus GluA1expression (*n*=9) groups (open: F(3, 61)=18.38, ***P<0.0001; close: F(3, 61)=11.22, ***P<0.0001, one-way analysis of variance; ***P<0.001, **P<0.01, Tukey's test *post hoc*). (**h**) Time spent in the open arm relative to total time in the open and the closed arms was compared. (*F*(3, 61)=16.38, ***P<0.0001; ***P<0.001, Tukey's test *post hoc*) (**i**) Number of entries into the open arm was measured (***P<0.0001, Kruskal–Wallis's test; ***P<0.01, Dunn's test *post hoc*). NS, not significant.

Finally, we evaluated the behavioural phenotypes of these AAV-injected mice. One of the major symptoms of FTLD is social impairment. We evaluated the social interaction of the AAV-injected mice by the four-session resident–intruder assay (Fig. 6a). Investigation time of the test mice on an unfamiliar wild-type intruder was not statistically different in the initial session. The interaction time of the control mice gradually decreased over the course of four sessions. However, FUS knockdown mice interacted with the intruder to a similar extent over the four sessions, indicating that the FUS knockdown animals are deficient in social behaviour.

### FUS knockdown elicits hyperactivity but not memory defects

Next, we performed the open field test to assess locomotor activity and anxiety in a novel environment. We found that FUS knockdown mice moved significantly longer distances in the total and the periphery of the open field than the controls (Fig. 6b), whereas there was no significant difference in centre duration or distance moved (Fig. 6b,c). Homecage activity, that is, basal motor activity in a familiar environment, was indistinguishable between the control and the knockdown groups (Supplementary Fig. 16). These results imply that FUS knockdown animals display a novelty-induced hyperactivity.

We then evaluated learning and memory of FUS knockdown mice by the novel object recognition test. Two different objects were presented on the first day; both the control and the FUS knockdown mice investigated two objects equally (Fig. 6d). The next day one object was replaced with a novel object. The control mice investigated the novel object for a longer period compared with the familiar object, indicative of normal object recognition memory. This behaviour was not affected by FUS depletion (Fig. 6e). However, the FUS knockdown mice investigated the objects overall for a longer period than the controls (Fig. 6f), which again indicates a novelty-induced hyperactivity. Although we evaluated learning and memory of the FUS knockdown mice by the fear conditioning assay, there was no detectable difference between the control and the FUS knockdown groups (Supplementary Fig. 17). These results indicate that hippocampal FUS depletion induces a novelty-induced hyperactivity, but does not vastly affect learning and memory.

### FUS depletion elicits dysinhibition that is rescued by GluA1 expression

Finally, we employed the elevated plus maze assay to evaluate behaviours such as anxiety and disinhibition. Control mice stayed in the closed arms for a longer period compared with the open arms, whereas FUS knockdown mice entered the open arms more often and spent dramatically more time there (Fig. 6g–i), indicating that FUS depletion elicits a disinhibition phenotype. Thus, hippocampal FUS knockdown leads to behavioural phenotypes related to novelty-induced hyperactivity and disinhibition, which might be responsible for social impairment, and are reminiscent of typical FTLD symptoms^21^.

These psychiatric phenotypes of the FUS knockdown mice, except for learning and memory, were remarkably similar to those of GluA1 KO mice^22^,^23^ (Supplementary Table 2). Last, we asked if the GluA1 reduction by FUS depletion in the hippocampus is responsible for the disinhibition phenotype in the elevated plus maze, as this behaviour was the most robust among the assays examined. When the mice were treated with AAV-shGluA1 (Supplementary Fig. 12), both open arm entry and duration were significantly exacerbated (Supplementary Fig. 18a–c), resembling the FUS knockdown phenotype. When exogenous GluA1 was introduced in FUS knockdown mice, the increased open arm entry and duration were ameliorated to the control level (Fig. 6g–i). We also observed a similar trend (*P*>0.05) for the rescue of social interaction defects and hyperactivity in the open field, whereas excessive object investigation was not rescued by GluA1 expression (Supplementary Fig. 19). shRNA-resistant FUS expression in FUS knockdown mice also rescued the disinhibition phenotype in the elevated plus maze. Together, these results indicate that behavioural aberrations caused by FUS depletion are, at least partly, mediated through the FUS-mediated control of GluA1 expression (Supplementary Table 2).

## Discussion

The work presented here adds another new insight into the molecular function of FUS, namely mRNA stability control. We show that FUS forms a protein complex with CPSF6, PABPC1 and PAN2, which strongly suggests that FUS modulates mRNA 3′-end processing. Similar to FUS, cytoplasmic polyadenylation element-binding protein CPEB, which is required for synaptic plasticity and learning and memory as well as meiotic progression during oogenesis, also interacts with both CPSFs and poly (A)-binding protein ePAB to regulate cytoplasmic polyadenylation of specific mRNAs^24^,^25^. Although CPSF6, the 68-kDa subunit of cleavage factor Im, is not specifically in the same complex as the one that CPEB interacts with, CPSF6 is also involved in mRNA export to the cytoplasm^26^.

We identified the deadenylase PAN2, which could be involved in poly (A) tail shortening of GluA1 mRNA. PAN2 is activated by PABP *in vitro*^27^. These data suggest that FUS might sequester PAN2 on the PABP from attacking the poly (A) tail. Upon FUS depletion, PAN2 is released from PABP and shortens the poly (A) tail. Alternatively, it is also possible that together with CPSF6, FUS might regulate 3′ polyadelyation site selection, as reported for TDP-43 (ref. 28). Our polyadenylation assay showed reduced signal intensity of the smear in spite that the PCR cycle was relatively saturated, which could suggest that an alternative polyadenylation site might be utilized in the FUS knockdown neurons. However, as FUS depletion reduces GluA1 mRNA in the cytoplasm and it interacts with cytoplasmic factors, PABPC1 and PAN2, we suggest it is more plausible that FUS regulates mRNA stability via the control of PAT at least for GluA1 mRNAs. Further biochemical analyses are needed to identify the mechanism by which FUS regulates mRNA polyadenylation. The identification of additional mRNAs whose stability is affected by FUS depletion is under investigation.

We show that FUS stabilizes GluA1 mature mRNA, which results in overall reduction in GluA1 protein level. Behavioural and morphological aberrations in FUS knockdown mice were rescued by exogenous GluA1, indicating that FUS-mediated GluA1 mRNA stability control is crucial for the phenotypes. Considering the variety of FTLD/ALS symptoms, it is certainly possible that some phenotypes may not be rescued by GluA1 expression due to the involvement of additional targets or mechanisms. Indeed, recent studies demonstrated that FUS R521C mutation found in familial ALS patients also elicits synaptic malfunctions likely via a toxic gain of function mechanism, which involve decreased interaction of FUS and histone deacetylase HDAC 1 and defective *Bdnf* splicing^7^,^29^. Nonetheless, the results presented here indicate the importance of this novel function of FUS on synaptic functions and some of FTLD-related behaviours.

To evaluate the intrinsic role of FUS in the brain, we stereotactically injected shRNA-expressing AAVs to deplete FUS *in vivo*. Surprisingly, we found that FUS knockdown specifically in the hippocampus resulted in FTLD-like behavioural phenotypes, such as disinhibition, hyperactivity and social interaction defects. These results might lead us to reconsider the importance of the brain regions affected in FTLD/ALS patients on physiological and behavioural manifestations of symptoms. Indeed, not only the frontal and temporal lobes, but also the hippocampus, is largely degenerated in a subpopulation of FTLD patients^18^,^30^. The hippocampus is the most affected region of the brain in Alzheimer's disease patients and it is believed that its major function is learning and memory. However, it is also involved in behaviours such as hyperactivity, emotion and anxiety^19^,^20^,^31^. Our result that FUS depletion in the hippocampus impacts hyperactivity and disinhibition, but not learning and memory, is interesting because the situation is similar in FTLD patients. GluA1 knockout mice show deficits in learning and memory. It may be possible that hyperactivity simply hindered our assays^32^. Another possibility is that FUS depletion affects only basal synaptic transmission and activity-induced synaptic plasticity, which is critical for learning and memory, might be rather intact (Supplementary Fig. 13). In any scenario in the future, it will be important to examine how FUS distinctively affects such animal behaviours and which downstream signalling pathways are specifically involved in these behavioural aberrations caused by FUS depletion.

## Methods

### Animals

All mice (C57BL/6J) were maintained in a temperature- (25 °C) and light-controlled (12-h light–dark cycle) environment (3–5 mice per cage). Animal protocols were approved for use by the Institutional Committee under Regulations on Animal Experiments at the Nagoya University.

### Neuron culture

Mouse cortical and hippocampal neuron cultures were prepared as described^33^. Cultured cortical and hippocampal neurons were maintained in Neurobasal medium supplemented with Glutamax (Life Technologies) and B27 supplements (Life Technologies). When needed, cultured neurons were treated with 1 μM cytosine arabinofuranoside to eliminate contaminating glial cells.

### DNA constructions

FUS knockdown lentiviral constructs were identical to those described previously^9^. The targeted sequences were 5′-GAGTGGAGGTTATGGTCAA-3′ (shFUS-1) and 5′-GAGTGGAGGTTATGGTCAA-3′ (shFUS-2). GluA1 knockdown lentiviral transfer vector was prepared on pLentiLox3.7-Syn as described^33^. The targeting sequence for GluA1, 5′-GCATTATCGACCATTACAA-3′, was chosen as described^34^. PAN2 knockdown lentiviral vector was constructed as described for GluA1 knockdown construct. Targeting sequences for PAN2 is 5′-CATCATGAGACAGACAAAT-3′. For lentiviral GluA1 expression, mouse GluA1 cDNA was cloned into the pFUGW lentiviral vector using *Xba*1/*Eco*R1 sites to make wild-type protein without tags.

For fluorescence reporter assay, the 3′ UTR sequence of GluA1 mRNA was amplified by RT–PCR using the primers 5′-AATTCCCCTGGAGCAGACAGGAAACCC-3′ and 5′-AGTCACTCGAGTAATGGGTCCACAGTGATTTAA-3′, and cloned into pcDNA-Venus-PEST (kind gift from N. Farny) at *Eco*R1/*Xho*1 sites.

For AAV-mediated transductions, AAV-s1 vector, a modified version of AAV-9-GFP (kind gift of K. Miyake), was used as a backbone for shRNA-mediated knockdown and for exogenous gene expression^35^. AAV-s1 has an AAV9 serotype backbone, which expresses shRNA under the H1 promoter as well as enhanced green fluorescent protein (EGFP) under the CAG promoter. The same shRNA sequences used for the lentivirus system mentioned above were cloned into AAV-s1 at Kpn1. For GluA1 expression, the CAG promoter in AAV-s1 was replaced with human Synapsin promoter at the *Nco*1/*Kpn*1 sites and mouse GluA1 cDNA was cloned into the Nhe1/Asc1 sites adapted to the 3′ end of the Synapsin promoter.

### Lentivirus production

Lentiviruses were produced using the vectors described above with packaging vectors pLP1, pLP2 and pVSVG in HEK293T cells (American Type Culture Collection). Transfection was performed in 6 or 10 cm dishes using Lipofectamine 2000 (Life Technologies) according to the manufacturer's instructions. Three hours after transfection, the medium was replaced with fresh DMEM with FBS and the virus-containing medium was collected and filtered 2 days later.

### Western blot

Cells scraped from neuron cultures or brain samples were homogenized with Brain IP buffer containing (in mM) 25 HEPES-KOH, pH 7.4, 150 NaCl, 5 MgCl_2_, 1 EDTA and 1% IGEPAL-CA630 supplemented with protease inhibitors (Roche), phosphatase inhibitors (Thermo Scientific) and RNase inhibitor (Takara) when needed. Homogenates were incubated on ice for 30 min and centrifuged at 15,000*g* for 10 min at 4 °C. The lysates were mixed with 4 × NuPAGE LDS sample buffer (Novex), heated at 70 °C for 5 min, and analysed by western blot using the antibodies listed in Supplementary Table 4. Band intensity was quantified by Multi Gauge 3.0 software (FujiFilm). All full blots are in Supplementary Fig. 20.

### Immunofluorescence

Cultured hippocampal neurons were processed for immunofluorescence as described previously^33^. Cultured hippocampal neurons grown on poly-L-lysine-coated coverclips were fixed in 4% paraformaldehyde for 20 min and permeabilized with 0.2% Trition X-100 diluted in PBS for 7 min. After blocking with 10% BSA diluted in PBS for 1 h at 37 °C in humidified chamber, neurons were processed with antibodies listed in Supplementary Table 4 and Fig. 1c. Neurons were imaged by a Zeiss LSM 710 confocal microscope using the same settings for all samples.

### Subcellular fractionation

Synaptoneurosomes and nuclear and cytoplasmic fractions were prepared from cultured cortical neurons as described^36^,^37^. For synaptoneurosome preparation, cultured cortical neurons (10 cm × 4) were washed with ice-cold PBS, scraped from the dish and spun at 1,000*g* for 5 min at 4 °C. The cell pellets were homogenized with pre-cooled 7 ml Dounce tissue grinder in 3 ml of homegenizing buffer containing (mM) 5 HEPES-KOH, pH 7.4, 320 sucrose, 1 EDTA, 1 mg ml^−1^ bovine serum albumin. The homogenate was centrifuged at 3,000*g* for 10 min at 4 °C. The supernatant was centrifuged at 14,000*g* for 12 min at 4 °C. The pellet was resuspended in 110 μl of Krebs–Ringer buffer containing (mM) 10 HEPES-KOH, pH 7.4, 140 NaCl, 5 KCl, 5 glucose, 1 EDTA. 90 μl of Percoll was added and spun at 14,000*g* for 2 min at 4 °C. The enriched synaptoneurosomes on the surface was recovered by aspirating the underlying solution and resuspended in 1 ml of Krebs–Ringer buffer. After a brief centrifugation at 14,000*g* for 1 min, the pellet containing synaptoneurosomes were resuspended in 200 μl of Krebs–Ringer buffer and used for western blot. For nuclear and cytoplasmic fractionation, cultured neurons (10 cm × 2) were washed with ice-cold PBS, scraped from the dish and collected by a brief centrifugation. The cells were lysed with 900 μl of buffer A containing (in mM) 10 HEPES-KOH, pH 7.4, 15 KCl, 2 MgCl_2_, 0.1 EDTA, 0.1% IGEPAL-CA630 and 1 dithiothreitol supplemented with protease inhibitors, phosphatase inhibitors and RNase inhibitor for 10 min on ice. The cytoplasmic extract was collected by centrifugation at 1,000*g* for 10 min at 4 °C. The pellet was resuspended in 300 μl of RIPA buffer containing (in mM) 25 Tris-HCl, pH 7.5, 150 NaCl, 1% IGEPAL-CA630, 1% sodium deoxycholate, 0.1% SDS supplemented with protease inhibitors, phosphatase inhibitors and RNase inhibitor, incubated for 30 min on ice, centrifuged at 18,000*g* for 20 min at 4 °C. The supernatant was collected as nuclear extract.

### Quantitative real-time PCR

RNA was prepared from neuron cultures using the RNeasy Mini kit (Qiagen) according the manufacturer's instructions. 500 ng of total RNA was used as template for reverse transcription using ImProm-II reverse transcriptase (Promega). qPCR was performed using primers listed in Supplementary Table 5 and iQ SYBR Green supermix (Bio-Rad) according to the manufacturer's instructions.

### Fluorescence *in situ* hybridization

FISH for GluA1 mRNA was performed as described previously^38^ with minor modifications. Primary neuron cultures were fixed by 4% paraformaldehyde in PBS for 20 min and permeabilized by 0.3% Triton X-100 diluted in PBS for 5 min. After three washes with PBS containing 5 mM MgCl_2_, the cells were equilibrated in 1 × saline sodium citrate (SSC) buffer for 10 min. *Escherichia coli* tRNA (1 μg) and single-stranded salmon sperm DNA (1 μg) were mixed, dried and resuspended in 15 μl of 80% formamide/1 × SSC, pH 7.0. The mixtures was heated at 95 °C for 5 min and added to15 μl of hybridization buffer (10% dextran sulfate, 2 × SSC, 4 mg ml^−1^ BSA and 10 mM sodium phosphate buffer, pH 7.0). The coverslips were incubated cell-side-down in 30 μl of pre-hybridization mix on parafilm at 37 °C in humidified chamber for 1 h. The digoxigenin-labelled anti-sense and sense control riboprobes for GluA1 mRNA were prepared as described^38^. The riboprobes (100 ng) were dried with the *E. coli* tRNA (10 μg) and single-stranded salmon sperm DNA (10 μg) and resuspended in 15 μl of 80% formamide/1 × SSC, pH 7.0. The mixture was heat denatured and mixed with 15 μl of hybridization buffer. The coverslips were incubated cell-side-down in 30 μl of a riboprobe-containing mixture overnight at 40 °C in humidified chamber. The coverslips were washed for 20 min in 40% formamide/1 × SSC at 40 °C, followed by three additional washes with 1 × SSC for 10 min at room temperature.

The cells were washed in TBS50 buffer (50 mM Tris-HCl, pH 7.4; 150 mM NaCl) for 5 min and permeabilized with TBS50 containing 0.3% Triton X-100 for 5 min. The cells were further incubated in immunofluorescence (IF) buffer (2% BSA; 0.1% Triton X-100 in TBS50) for 5 min and incubated in blocking buffer (2%BSA; 2% fetal bovine serum; 0.1% Triton X-100) for 30 min at room temperature. Subsequently, the cells were incubated with a primary mouse anti-digoxigenin antibody (1:100; Roche Applied Science) for 1 h at room temperature. After the wash with IF buffer for 10 min, the cells were incubated with AlexaFluor 555-conjugated goat anti-mouse antibody (Invitrogen) diluted in IF buffer (1:1,000) for 1 h at room temperature. After three washes with IF buffer, the coverslips were mounted with DAPI fluoromount-G (Southern Biotech).

Samples were imaged on a Zeiss LSM 710 confocal microscope using the same settings for all samples. FISH signals were quantified by Zen software (Zeiss) in the cell body compartment and in the 70 μm of the distal dendritic segments starting from 30 μm from the edge of the soma up to 100 μm.

### Immunoprecipitation

The total cell lysates prepared as above were mixed with 1–2 μg of antibody and rotated at 4 °C for 1 h. Protein G dynabeads (Invitrogen) was added to the mixture and further incubated at 4 °C for 2 h. After the beads were washed with Brain IP buffer four times, bound protein and RNA were analysed as below. To examine protein–protein interactions (Fig. 3e), Brain IP buffer was supplemented with either RNase inhibitor (Takara) or RNase A (Qiagen) throughout the procedure. The beads were directly mixed with 2 × NuPAGE LDS-PAGE sample buffer (Novex) containing β-mercaptoethanol, heated at 70 °C for 10 min and analysed by western blotting. To analyse the bound RNA (Fig. 3a), the buffer was supplemented with RNase inhibitor (Takara). Equal amounts of TE and phenol were added to the beads and vigorously vortexed. The RNA collected in the aqueous fraction was chloroform-washed and pelleted by ethanol precipitation with glycogen. The RNA was resuspended in distilled water and analysed by qRT–PCR.

### Biotinylated RNA pulldown assay

To assess the interaction of GluA1 mRNA 3′ UTR with endogenous FUS, a biotinylated RNA pull-down assay was performed as described^39^. GluA1 3′UTR sequence was amplified by RT–PCR using primers 5′-TAATACGACTCACTATAGGGAAGAAGTTACCTTGTATTATGTAT-3′ and 5′-TAATGGGTCCACAGTGATTTAA-3′. PCR product was purified by PCR purification kit (Qiagen) and used as template for *in vitro* transcription reaction using T7 RNA polymerase (Takara) with biotin RNA labelling mix (Roche). The biotinylated RNA was incubated with streptoavidin dynabeads in Brain IP buffer. The lysate from cortical neuron cultures prepared as described above was added to the RNA-beads suspension. Two hours after the incubation at room temperature, the beads were washed with Brain IP buffer five times and mixed with 2 × NuPAGE LDS-PAGE sample buffer (Novex) containing β-mercaptoethanol. Total lysate and the bound proteins were analysed by western blot.

### Fluorescence reporter assay

To examine the involvement of GluA1 3′ UTR sequence in its expression, a fluorescence reporter assay was performed. The GluA1 3′ UTR (or a control SCNA1 3′ UTR) was cloned onto a variant of yellow fluorescent protein, Venus, fused with a PEST sequence that increases the sensitivity of the reporter by destabilizing the protein fused to it. The reporter constructs were co-transfected into Neuroblastoma Neuro2A cells with the appropriate lentiviral shRNA constructs by Lipofectamine 2000 (Invitrogen) according to the manufacturer's instructions. 24 h post transfection, bright-field and fluorescence images were obtained using a fluorescence microscope (Olympus). The level of fluorescence was measured relative to equal number of cells by Image J software.

### Polyadenylation assay

GluA1 mRNA PAT was assayed by Poly (A) tail length assay kit (Affymetrix) according to the manufacturer's instructions with slight modifications. RNA was purified from cytoplasmic and nuclear fractions prepared as above. The following GluA1-specific primers were used: 5′-AAATATTTGGGGTAGGGATTTC-3′ and 5′-TAATGGGTCCACAGTGATTTAA-3′.

### AAV production

AAVs were produced and purified as described previously^40^. Subconfluent AAV-human embryonic kidney 293 cells (AAV-HEK293) were transiently transfected with the vector plasmid and two helper plasmids, pAAV2-9 (kind gift of Dr James M. Wilson) and pHelper (kind gift of Dr K. Miyake), using the calcium phosphate co-precipitation method. Seventy-two hours after transfection, cells were collected by low-speed centrifugation, resuspended in Tris-buffered saline (100 mM Tris-HCl (pH 8.0), 150 mM NaCl) and lysed by three freeze and thaw cycles. Lysate was treated with Benzonase (200 U ml^−1^) with 5 mM MgCl_2_ and the reaction was stopped with 6.5 mM EDTA. AAV vectors were concentrated by two-tier CsCl gradient centrifugation (1.25 and 1.50 g ml^−1^ of CsCl in HEN buffer (50 mM HEPES (pH7.4), 150 mM NaCl, 25 mM EDTA)) for 3 h (16 °C, 25,000 r.p.m., SW28 (Beckman)). Viral-rich fraction was collected by measuring refractive index (1.371–1.380). The collected solution was purified using continuous CsCl gradients centrifugation (1.39 g ml^−1^ of CsCl in HEN buffer) for 16 h (18 °C, 38,000 r.p.m., SW40 (Beckman)), and a part of refractive index 1.371–1.380 was collected, dialysized against thre changes of HEPES-buffered saline (HEPES 40 mM (pH 7.4), 150 mM NaCl), concentrated using filter and stored at −80 °C. Titres of the viral stocks were determined by qPCR of the vector stocks.

### Stereotactic injection of AVV

AAVs were bilaterally injected into the hippocampus of 6-week-old male mice as described^41^. 1 μl of viral titre solution was injected bilaterally into the hippocampus (1.58 mm posterior to bregma, 1.50 mm lateral to midline and 1.80 mm below the skull surface) at a flow rate of 0.5 μl min^−1^. After the injection, the mice recovered for 6 weeks.

### Hippocampal slice preparation

Hippocampal slices were prepared from the mice aged 12–13 weeks. Mice were decapitated under deep anaesthesia with ethyl ether. Brains were quickly removed, and coronal 350-mm-thick slices were cut from the hippocampus using a vibratome in ice-cold modified artificial cerebrospinal fluid (ACSF) containing 206 mM sucrose, 5 mM KCl, 8 mM MgCl_2_, 1.25 mM KH_2_PO_4_, 1 mM CaCl_2_, 26 mM NaHCO_3_ and 10 mM D-glucose. ACSF was gassed with 95% O_2_/5% CO_2_ and the pH was adjusted to 7.4. Slices were maintained for at least 1.5 h at room temperature (26–28 °C) in an incubation chamber containing gassed standard ACSF containing 128 mM NaCl, 5 mM KCl, 1.3 mM MgSO_4_, 1.25 mM KH_2_PO_4_, 2.41 mM CaCl_2_, 26 mM NaHCO_3_ and 10 mM D-glucose.

### Electrophysiology

A single hippocampal slice was transferred to the recording chamber where it was superfused continuously with gassed standard ACSF at a rate of 2–2.5 ml min^−1^ at room temperature. For whole-cell patch-clamp recordings from CA1 pyramidal neurons of the hippocampal slice, a patch electrode was filled with a pipette solution containing (in mM) 140 cesium gluconate, 10 NaCl, 2 MgCl_2_, 1 EGTA, 10 HEPES, 3 Mg-ATP, 0.3 Na-GTP (pH 7.2), with 6–8 MΩ of resistance. CA1 pyramidal neurons were imaged with an IR-DIC optics (BX51WI) with × 20 water immersion objective lens (OLYMPUS). Whole-cell patch-clamp recordings from cultured cortical neurons were made at room temperature with an external solution containing (in mM) 140 NaCl, 3.5 KCl, 2 CaCl_2_, 2 MgCl_2_,10 HEPES and 20 D-glucose (pH 7.4) and a pipette solution containing (in mM) 140 K gluconate, 10 KCl, 2 MgCl_2_, 0.2 EGTA, 10 HEPES, 3 Mg-ATP, 0.3 Na-GTP (pH 7.2), with 6–8 MΩ of resistance. Holding potentials were compensated for the junction potential between the pipette solution and the external solution (or the standard ACSF). AMPA receptor-mediated mEPSCs were recorded in voltage-clamp mode at a membrane potential of −70 mV and in the presence of voltage-dependent Na^+^ channel blocker (0.5 mM TTX) and GABA_A_ receptor antagonist (50 mM picrotoxin). Access resistance was monitored continuously during the experiment, and the obtained data were discarded if the access resistance fluctuated over 20%. Signals were amplified and filtered at 5 kHz with an amplifier (Axopatch 200B, Axon Instruments). Data acquisition and analysis were performed using pCLAMP 9.0 software (Axon Instruments). Miniature EPSCs were identified by setting the event detection threshold at 5 pA and by checking that the events had faster rising times than decay times.

### Golgi-Cox staining

The whole brain was dissected from the AAV-injected mice and subjected to the FD Rapid GolgiStain kit (FD Neuro Technologies) according to the manufacturer's instructions. In the CA1 apical dendrite, 100-μm-long dendritic segments from the first branch were analysed to quantify spine density and spine morphology. Unbranched spines that had more than 1.5-fold larger head diameter than neck diameter were defined as mushroom-shaped spines.

### Behavioural assays

Behavioural assays were conducted on the mice aged 12–15 weeks during light cycle. Mice were examined in the homecage activity test^42^, open field test, elevated plus maze test, novel object recognition test, social interaction (resident-intruder) test and fear conditioning test^43^,^44^, in the same experimental settings as described previously. Mice were used for each behavioural test only once. Outliers were excluded based on the Grubb's test.

### Open field test

Mice were placed in the centre of the arena and were allowed to explore the open field (diameter: 60 cm, height: 35 cm) for the following 5 min under moderately light conditions (80 lx), while their activity was measured automatically using the Ethovision automated tracking programme (Brainscience Idea Co., Ltd). The open field was divided into an inner circle (diameter: 40 cm) and an outer area surrounding the inner circle. The movement of mice was measured via a camera mounted above the open field. Measurements included distance and time spent in the inner and outer sections.

### Elevated-plus maze test

The elevated-plus maze consisted of two open (25 × 8 × 0.5 cm^3^) and two closed (25 × 8 × 20 cm^3^) arms emanating from a common central platform (8 × 8 cm^2^) to form a plus shape. The entire apparatus was elevated to 50 cm above floor level under moderately bright conditions (170 lux). The test began by placing a mouse on the central platform of the maze facing an open arm. An arm entry was defined as all four paws in the arm. The duration of time spent in an arm and number of arm entries was measured for 5 min.

### Novel object recognition test

Mice were individually habituated to an open-box (30 × 30 × 35 cm^3^) for 3 days. During the training session, two novel objects were placed in the open field and the animals were allowed to explore for 10 min under moderately light conditions (10 lx). The time spent exploring each object was recorded. During retention sessions, the animals were placed back into the same box 24 h after the training session, one of the familiar objects used during training was replaced by a novel object, and the mice were allowed to explore freely for 5 min. The preference index in the retention session, the ratio of the amount of time spent exploring the novel object over the total time spent exploring both objects, was used to measure cognitive function. In the training session, the preference index was calculated as the ratio of time spent exploring the object that was replaced by a novel object in the retention session, to the total exploration time.

### Social interaction (resident–intruder) test

Animals were individually housed in a home cage (29 × 18 × 12 cm^3^) for 2 days before the trial. We used sex- and age-matched C57BL/6J mice that had not previously shown aggressive behaviour, as intruders. During the first trial (5 min duration), an intruder mouse was introduced into the resident's home cage under moderate light (60 lux). The duration of social interaction (close following, inspection, anogenital sniffing and other social body contacts, except aggressive behaviour) were analysed. Four trials were performed with an inter-trial interval of 30 min, and social behaviour was analysed using the same intruder mouse.

### Fear conditioning test

During the conditioning phase, each mouse was placed in a training chamber (30 × 30 × 40 cm^3^) equipped with a metal floor, and a 15-s white noise tone (85 dB) was delivered (conditioned stimulus). During the last 5 s of the tone stimulus, a foot shock of 0.8 mA was delivered through a shock generator as an unconditioned stimulus (Brainscience Idea). This procedure was repeated four times at 15-s intervals. Twenty-four hours after conditioning, the context-dependent test was performed. For the context-dependent test, each mouse was placed in the training chamber, and the freezing response was measured for 2 min in the absence of the conditioned stimulus. Tone-dependent testing was performed 4 h after the context-dependent test. For the tone-dependent test, the freezing response was measured in a standard transparent rectangular rodent cage (25 × 30 × 18 cm^3^) for 1 min in the presence of a continuous-tone stimulus identical to the conditioned stimulus using mice that had been subjected to the context-dependent test.

### Statistics

For western blot and qRT–PCR, the data were obtained from at least three independent experiments. The data were presented as relative value to control. For mRNA decay assay, the data for shCtrl and shFUS were each normalized to time 0 (pretreatment) and the data at 24 h were used for statistical analysis. For quantification of the FUS-bound mRNAs (Fig. 3a), representative data from triplicate experiments is shown, as the IP efficiency for each transcript varied from experiment to experiment, while the pattern of IP efficiency between primary and mature transcripts remained same in all experiments. For polyadenylation assay (Fig. 3d), representative gel image from three independent experiments is shown, as the length of poly (A) smear varied from experiment to experiment, while the tendency that the FUS knockdown has shorter poly (A) tail remained same in all experiments. Representative data from three independent experiments were shown for IP (Fig. 3b, e, and Supplementary Fig. 7). No statistical analysis was used to predetermine the sample sizes used for experiments; however, our sample sizes are similar to those reported previously. No randomization was used for data collection and analysis. All data were analysed by Graphpad Prism software. Normality was tested by the KS normality test. For statistical analysis of two groups, the paired or the unpaired *t-*test was used as described in figure legend. When the data were not normally distributed, the Mann–Whitney's *U*-test was used (Fig. 4 and Supplementary Fig. 17a, c). In experiments with more than two groups, one-way analysis of variance *post hoc* Tukey's multiple comparison test was used. When the data were not normally distributed, the Kruskal–Wallis test *post hoc* Dunn's test was used (Fig. 6i). In all experiments, the data were expressed as mean±s.e.m. and the determination of statistical significance was set to *P*<0.05. For the experiments using cell cultures, the *n* numbers refer to the number of experiments from separate cultures, unless otherwise stated. For the experiments using mice, the *n* numbers refer to the number of mice used for experiments, with the exception of spine analysis (Fig. 5h, i and Supplementary Fig. 15b), where *n* refers to the number of dendrites from three mice. The data collection and analysis were not performed blind, with exceptions of behavioural experiments, where two experimenters collected and analysed the data separately.

## Additional information

**How to cite this article:** Udagawa, T. *et al.* FUS regulates AMPA receptor function and FTLD/ALS-associated behaviour via GluA1 mRNA stabilization. *Nat. Commun.* 6:7098 doi: 10.1038/ncomms8098 (2015).

## Supplementary Material

Supplementary Information Supplementary Figures 1-20 and Supplementary Tables 1-4

## References

[b1] Crozat A., Åman P., Mandahl N. & Ron D. Fusion of CHOP to a novel RNA-binding protein in human myxoid liposarcoma. Nature 363, 640–644 (1993). 8510758 10.1038/363640a0

[b2] Vance C. *et al.* Mutations in FUS, an RNA processing protein, cause familial amyotrophic lateral sclerosis type 6. Science 323, 1208–1211 (2009). 19251628 10.1126/science.1165942 PMC4516382

[b3] Kwiatkowski T. J. *et al.* Mutations in the FUS/TLS gene on chromosome 16 cause familial amyotrophic lateral sclerosis. Science 323, 1205–1208 (2009). 19251627 10.1126/science.1166066

[b4] Deng H.-X. *et al.* FUS-immunoreactive inclusions are a common feature in sporadic and non-SOD1 familial amyotrophic lateral sclerosis. Ann. Neurol. 67, 739–748 (2010). 20517935 10.1002/ana.22051 PMC4376270

[b5] Munoz D. G. *et al.* FUS pathology in basophilic inclusion body disease. Acta Neuropathol. 118, 617–627 (2009). 19830439 10.1007/s00401-009-0598-9

[b6] Ling S., Polymenidou M. & Cleveland D. W. Review converging mechanisms in ALS and FTD: disrupted RNA and protein homeostasis. Neuron 79, 416–438 (2013). 23931993 10.1016/j.neuron.2013.07.033 PMC4411085

[b7] Qiu H. *et al.* ALS-associated mutation FUS-R521C causes DNA damage and RNA splicing defects. J. Clin. Invest. 124, 981–999 (2014). 24509083 10.1172/JCI72723 PMC3938263

[b8] Lagier-Tourenne C. *et al.* Divergent roles of ALS-linked proteins FUS/TLS and TDP-43 intersect in processing long pre-mRNAs. Nat. Neurosci. 15, 1488–1497 (2012). 23023293 10.1038/nn.3230 PMC3586380

[b9] Ishigaki S. *et al.* Position-dependent FUS-RNA interactions regulate alternative splicing events and transcriptions. Sci. Rep 2, 529 (2012). 22829983 10.1038/srep00529 PMC3402842

[b10] Rogelj B. *et al.* Widespread binding of FUS along nascent RNA regulates alternative splicing in the brain. Sci. Rep 2, 603 (2012). 22934129 10.1038/srep00603 PMC3429604

[b11] Fujii R. & Takumi T. TLS facilitates transport of mRNA encoding an actin-stabilizing protein to dendritic spines. J. Cell Sci. 118, 5755–5765 (2005). 16317045 10.1242/jcs.02692

[b12] Yasuda K. *et al.* The RNA-binding protein Fus directs translation of localized mRNAs in APC-RNP granules. J. Cell Biol. 203, 737–746 (2013). 24297750 10.1083/jcb.201306058 PMC3857475

[b13] Fujioka Y. *et al.* FUS-regulated region- and cell-type-specific transcriptome is associated with cell selectivity in ALS/FTLD. Sci. Rep 3, 2388 (2013). 23925123 10.1038/srep02388 PMC3737506

[b14] Meijer H. A. & de Moor C. H. Fractionation of mRNA based on the length of the poly(A) tail. Methods Mol. Biol. 703, 123–135 (2011). 21125487 10.1007/978-1-59745-248-9_9

[b15] Vance C. *et al.* ALS mutant FUS disrupts nuclear localization and sequesters wild-type FUS within cytoplasmic stress granules. Hum. Mol. Genet 22, 2676–2688 (2013). 23474818 10.1093/hmg/ddt117 PMC3674807

[b16] Huganir R. L. & Nicoll R. A. AMPARs and synaptic plasticity: the last 25 years. Neuron 80, 704–717 (2013). 24183021 10.1016/j.neuron.2013.10.025 PMC4195488

[b17] Lu W. & Roche K. W. Posttranslational regulation of AMPA receptor trafficking and function. Curr. Opin. Neurobiol. 22, 470–479 (2012). 22000952 10.1016/j.conb.2011.09.008 PMC3279598

[b18] Hornberger M. *et al.* *In vivo* and post-mortem memory circuit integrity in frontotemporal dementia and Alzheimer's disease. Brain 135, 3015–3025 (2012). 23012333 10.1093/brain/aws239

[b19] Barkus C. *et al.* Hippocampal NMDA receptors and anxiety: at the interface between cognition and emotion. Eur. J. Pharmacol. 626, 49–56 (2010). 19836379 10.1016/j.ejphar.2009.10.014 PMC2824088

[b20] Bannerman D. M. *et al.* The time course of the hyperactivity that follows lesions or temporary inactivation of the fimbria-fornix. Behav. Brain Res. 120, 1–11 (2001). 11173080 10.1016/s0166-4328(00)00354-5

[b21] Rascovsky K. *et al.* Sensitivity of revised diagnostic criteria for the behavioural variant of frontotemporal dementia. Brain 134, 2456–2477 (2011). 21810890 10.1093/brain/awr179 PMC3170532

[b22] Feyder M., Wiedholz L., Sprengel R. & Holmes A. Impaired associative fear learning in mice with complete loss or haploinsufficiency of AMPA GluR1 receptors. Front. Behav. Neurosci. 1, 4 (2007). 18958186 10.3389/neuro.08.004.2007 PMC2525858

[b23] Vekovischeva O. Y. *et al.* Reduced aggression in AMPA-type glutamate receptor GluR-A subunit-deficient mice. Genes Brain Behav. 3, 253–265 (2004). 15344919 10.1111/j.1601-1848.2004.00075.x

[b24] Kim J. H. & Richter J. D. RINGO/cdk1 and CPEB mediate poly(A) tail stabilization and translational regulation by ePAB. Genes Dev. 21, 2571–2579 (2007). 17938241 10.1101/gad.1593007 PMC2000322

[b25] Darnell J. C. & Richter J. D. Cytoplasmic RNA-binding proteins and the control of complex brain function. Cold Spring Harb. Perspect. Biol 4, a012344 (2012). 22723494 10.1101/cshperspect.a012344 PMC3405866

[b26] Ruepp M.-D. *et al.* Mammalian pre-mRNA 3′ end processing factor CF Im68 functions in mRNA export. Mol. Biol. Cell 20, 5211–5223 (2009). 19864460 10.1091/mbc.E09-05-0389 PMC2793296

[b27] Uchida N., Hoshino S.-I. & Katada T. Identification of a human cytoplasmic poly(A) nuclease complex stimulated by poly(A)-binding protein. J. Biol. Chem. 279, 1383–1391 (2004). 14583602 10.1074/jbc.M309125200

[b28] Bembich S. *et al.* Predominance of spliceosomal complex formation over polyadenylation site selection in TDP-43 autoregulation. Nucleic Acids Res. 42, 3362–3371 (2014). 24369426 10.1093/nar/gkt1343 PMC3950720

[b29] Wang W.-Y. *et al.* Interaction of FUS and HDAC1 regulates DNA damage response and repair in neurons. Nat. Neurosci. 16, 1383–1391 (2013). 24036913 10.1038/nn.3514 PMC5564396

[b30] Brettschneider J. *et al.* Stages of pTDP-43 pathology in amyotrophic lateral sclerosis. Ann. Neurol. 74, 20–38 (2013). 23686809 10.1002/ana.23937 PMC3785076

[b31] Kheirbek M. A. *et al.* Differential control of learning and anxiety along the dorsoventral axis of the dentate gyrus. Neuron 77, 955–968 (2013). 23473324 10.1016/j.neuron.2012.12.038 PMC3595120

[b32] Sanderson D. J. *et al.* Deletion of the GluA1 AMPA receptor subunit impairs recency-dependent object recognition memory. Learn. Mem 18, 181–190 (2011). 21378100 10.1101/lm.2083411 PMC3056514

[b33] Udagawa T. *et al.* Bidirectional control of mRNA translation and synaptic plasticity by the cytoplasmic polyadenylation complex. Mol. Cell 47, 253–266 (2012). 22727665 10.1016/j.molcel.2012.05.016 PMC3408552

[b34] Lin C. & Lee E. JNK1 inhibits GluR1 expression and GluR1-mediated calcium influx through phosphorylation and stabilization of Hes-1. J. Neurosci 32, 1826–1846 (2012). 22302822 10.1523/JNEUROSCI.3380-11.2012 PMC6703358

[b35] Noro T. *et al.* Adeno-associated viral vector-mediated expression of endostatin inhibits tumor growth and metastasis in an orthotropic pancreatic cancer model in hamsters. Cancer Res. 64, 7486–7490 (2004). 15492274 10.1158/0008-5472.CAN-03-1296

[b36] Arguelles S. *et al.* Molecular control of the amount, subcellular location, and activity state of translation elongation factor 2 in neurons experiencing stress. Free Radic. Biol. Med. 61C, 61–71 (2013). 23542375 10.1016/j.freeradbiomed.2013.03.016 PMC3772990

[b37] Napoli I. *et al.* The fragile X syndrome protein represses activity-dependent translation through CYFIP1, a new 4E-BP. Cell 134, 1042–1054 (2008). 18805096 10.1016/j.cell.2008.07.031

[b38] La Via L. *et al.* Modulation of dendritic AMPA receptor mRNA trafficking by RNA splicing and editing. Nucleic Acids Res. 41, 617–631 (2013). 23166306 10.1093/nar/gks1223 PMC3592400

[b39] López de Silanes I., Zhan M., Lal A., Yang X. & Gorospe M. Identification of a target RNA motif for RNA-binding protein HuR. Proc. Natl Acad. Sci. USA 101, 2987–2992 (2004). 14981256 10.1073/pnas.0306453101 PMC365732

[b40] Okada T. *et al.* Scalable purification of adeno-associated virus serotype 1 (AAV1) and AAV8 vectors, using dual ion-exchange adsorptive membranes. Hum. Gene Ther. 20, 1013–1021 (2009). 19534598 10.1089/hum.2009.006

[b41] Udagawa T. *et al.* Genetic and acute CPEB1 depletion ameliorate fragile X pathophysiology. Nature Med 19, 1473–1477 (2013). 24141422 10.1038/nm.3353 PMC3823751

[b42] Miyazaki Y. *et al.* Viral delivery of miR-196a ameliorates the SBMA phenotype via the silencing of CELF2. Nature Med 18, 1136–1141 (2012). 22660636 10.1038/nm.2791

[b43] Kuroda K. *et al.* Behavioral alterations associated with targeted disruption of exons 2 and 3 of the Disc1 gene in the mouse. Hum. Mol. Genet 20, 4666–4683 (2011). 21903668 10.1093/hmg/ddr400

[b44] Ibi D. *et al.* Neonatal polyI: C treatment in mice results in schizophrenia-like behavioral and neurochemical abnormalities in adulthood. Neurosci. Res. 64, 297–305 (2009). 19447299 10.1016/j.neures.2009.03.015

