# Supplementary material for: FUS regulates AMPA receptor function and FTLD/ALS-associated behaviour via GluA1 mRNA stabilization
Source: Nat Commun. 2015 May 13;6:7098. doi: 10.1038/ncomms8098 (PMC4479014; doi:10.1038/ncomms8098)
Supplement: Supplementary Information — Supplementary Figures 1-20 and Supplementary Tables 1-4 [file ncomms8098-s1.pdf]

Supplementary Figure 1

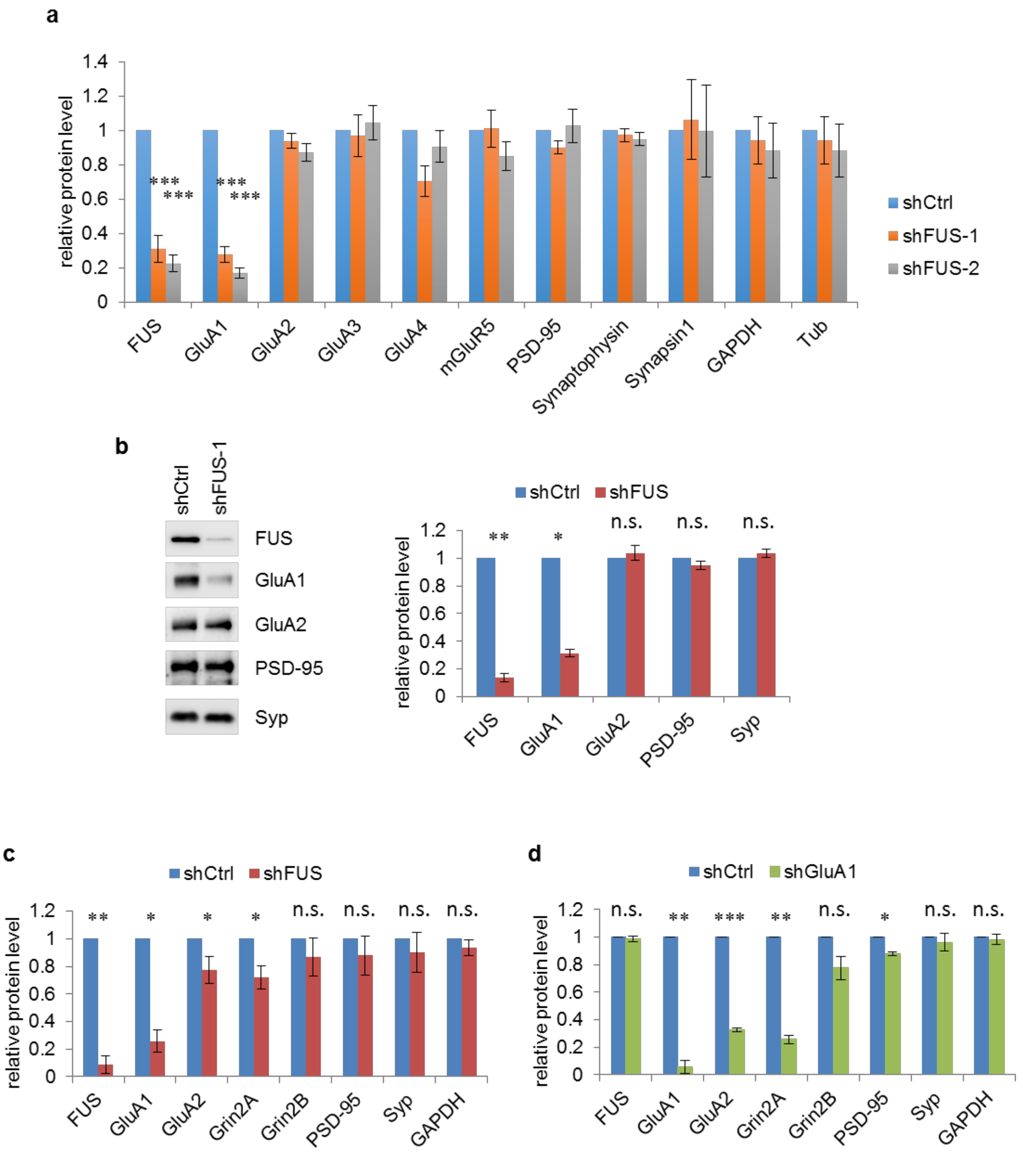

Supplementary Figure 1: FUS depletion down-regulates the expression of GluA1 and other post-synaptic proteins (**a**) Quantification of western blot in Fig. 1a (FUS: $n = 3$, $F(2, 6) = 62.10$, $P < 0.0001$, one-way ANOVA; ***$P < 0.001$, GluA1: $n = 4$, $F(2, 9) = 298.4$, $P < 0.0001$,

one-way ANOVA; ***$P < 0.001$, Tukey's test *post hoc*) (**b**) Mouse cultured hippocampal neurons, DIV10, were infected with lentiviruses expressing shRNA against FUS (shFUS) or scrambled shRNA (shCtrl). Ten days post infection the lysates were prepared, analyzed by western blot, and detected proteins were quantified ($n = 3$ each, FUS: $t = 29.18$, **$P = 0.012$; GluA1: $t = 25.10$, **$P = 0.016$, paired *t*-test). (**c**) Cultured hippocampal neurons, DIV7, were infected with shCtrl or shFUS, and incubated for 15-19 days. The lysates were western blotted and quantified for the indicated proteins ($n = 3$ each, FUS: $t = 14.89$, **$P = 0.0045$; GluA1: $t = 9.393$, *$P = 0.011$; GluA2: $t = 3.422$, *$P = 0.042$; Grin2A: $t = 4.773$, **$P = 0.041$, paired *t*-test). (**d**) Cultured cortical neurons, DIV10, were infected with lentiviruses expressing shCtrl or shRNA against GluA1 (shGluA1). Seven days post infection the lysates were prepared and analyzed by western blot ($n = 3$ each, GluA1: $t = 20.98$, **$P = 0.0023$; GluA2: $t = 55.58$, ***$P = 0.0003$; Grin2A: $t = 23.82$, **$P = 0.0018$; PSD-95: $t = 6.266$, *$P = 0.025$, paired *t*-test). (**d**) Immunohistochemistry for GluA1 in the brain of the control and two FTLD/ALS-FUS patients. The granule cell layer of the hippocampus near the CA-2 region is presented. The expression of GluA1 within the dendrites and neuronal soma of the patients appears to be weaker than that of control material. Anti-GluA1 immunohistochemistry counterstained by hematoxylin-eosin staining. Original magnifications are ×200 and ×400.

Supplementary Figure 2

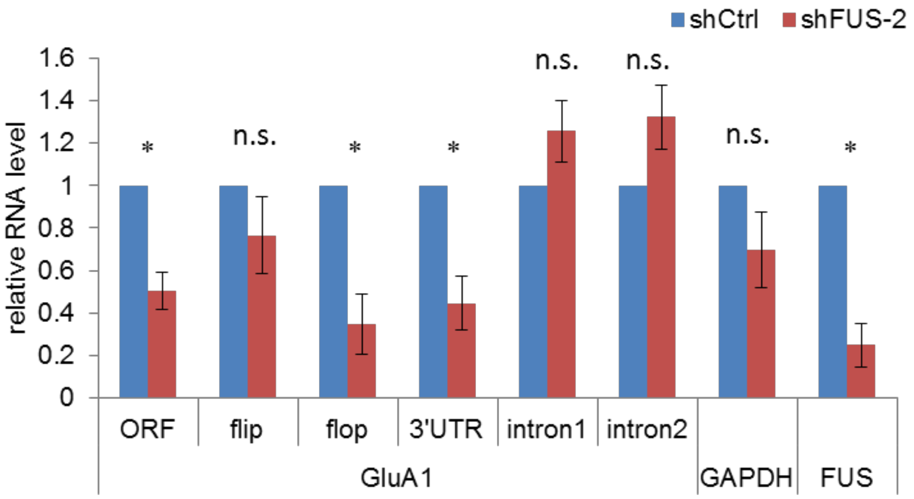

Supplementary Figure 2: Second shRNA against FUS reduces GluA1 mature mRNA level. Cultured cortical neurons were transduced by lentiviruses expressing shCtrl or shFUS-2. The transcript levels were analyzed as described in Fig. 2b legend (n = 3 each, ORF: t = 5.595, *P = 0.031; flop: t = 4.582, *P = 0.045; 3'UTR: t = 4.404, *P = 0.048; FUS: t = 7.461, **P = 0.018, paired t-test).

Supplementary Figure 3

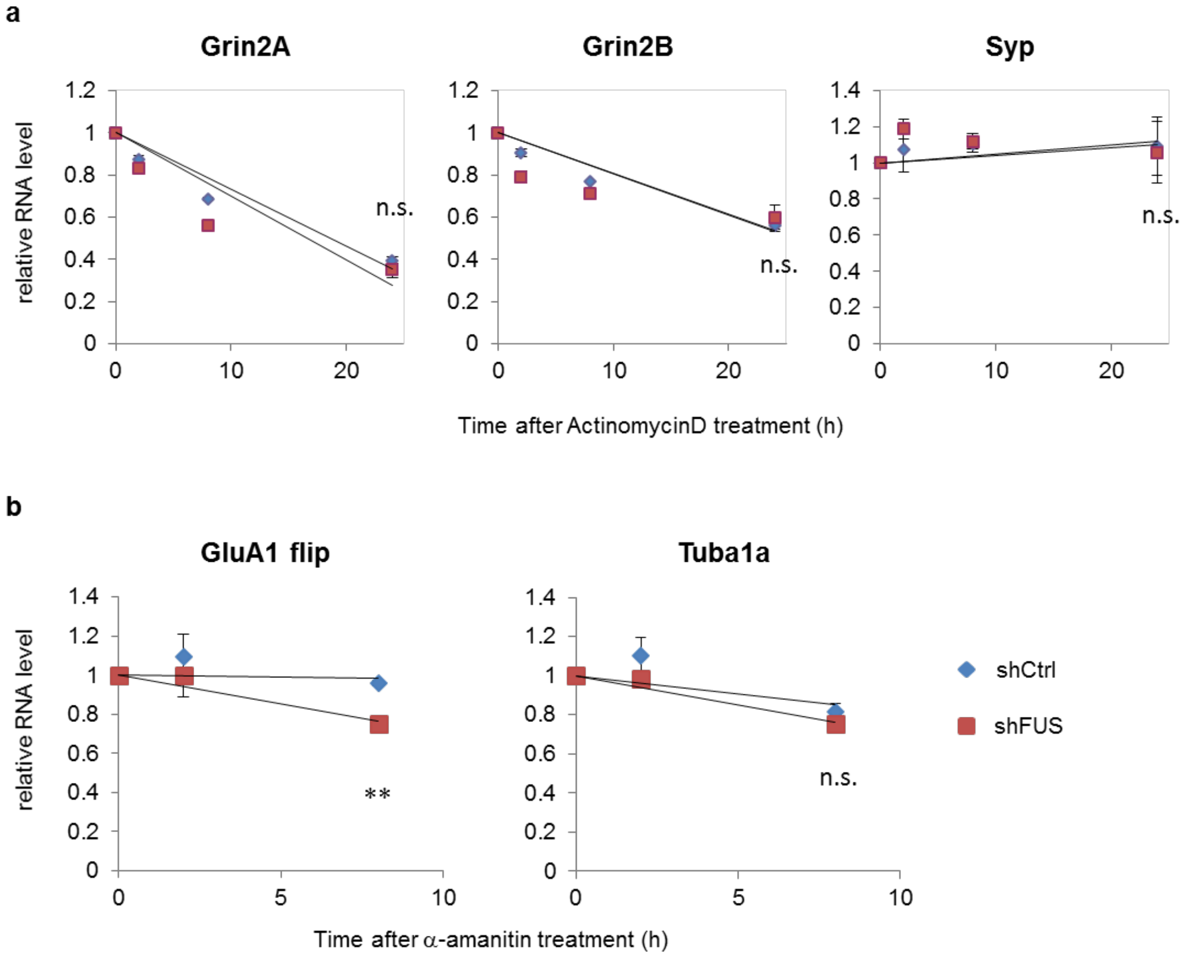

Supplementary Figure 3: GluA1 mRNA is destabilized by FUS depletion. (**a**) The stability of mRNA encoding Grin2A, Grin2B, and Synaptophysin was measured as in Fig. 2d. (Grin2A: t = 1.008, P = 0.3706; Grin2B: t = 0.5542, P = 0.6090; Synaptophysin: t = 0.1481, P = 0.8894, unpaired t-test) (**b**) The stability of GluA1 flip and Tuba1a mRNA was examined by the 40 μg/ml α-amanitin as described for Actinomycin D in Fig. 2d (n = 3 each, GluA1 flip: t = 8.229, **P = 0.0012; Tuba1a: t = 2.502, P = 0.067, unpaired t-test, at 8 h)

Supplementary Figure 4

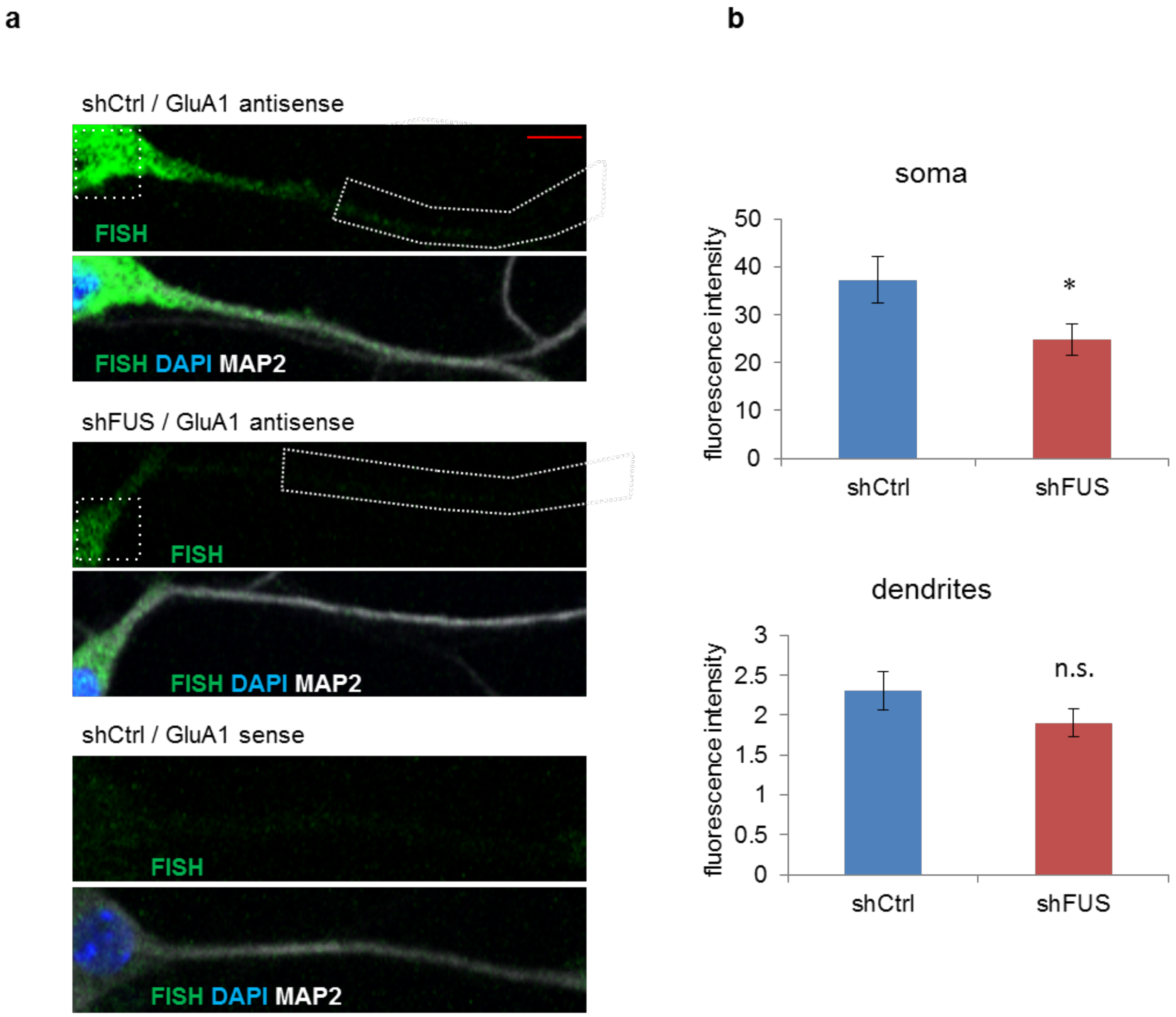

Supplementary Figure 4: FUS depletion does not affect GluA1 mRNA transport into dendrites. (**a**) Representative images of fluorescent *in situ* hybridization (FISH) using GluA1 antisense or sense probe and MAP2 immunostaining together with DAPI staining in the shCtrl and shFUS hippocampal neurons. Scale bar; 10 μm. (**b**) FISH signal intensities in the cell bodies and the dendrites (enclosed) were quantified as described in Online Methods. (soma: n = 22 each from 3 separate cultures, *P = 0.035, Mann-Whitney's U test; dendrites: n = 20 each, from 3 separate cultures, t = 1.317, P = 0.19, unpaired t-test)

Supplementary Figure 5

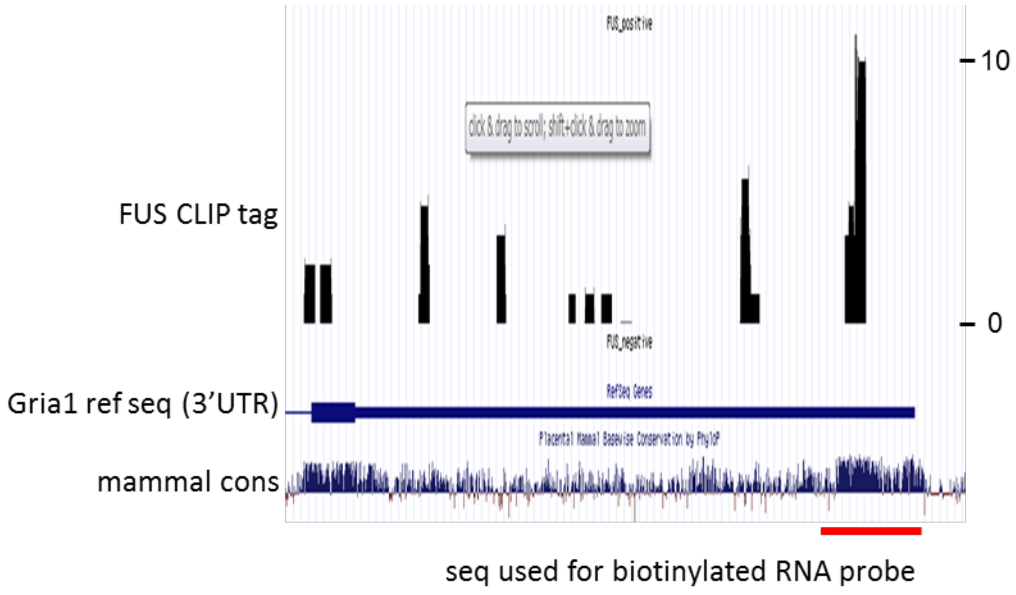

Supplementary Figure 5: CLIP tag enrichments in the 3' UTR of GluA1 mRNA. HITS-CLIP data (GSE37190) was uploaded on the UCSC genome browser and the image focusing on the GluA1 (Gria1) 3'UTR was extracted. Number of CLIP tags on each nucleotide of the GluA1 3' UTR sequences is presented as black bar on top and the conservation level of the sequences among mammalian species is indicated on the bottom. Red bar indicates the region used for the RNA in Fig. 3B.

Supplementary Figure 6

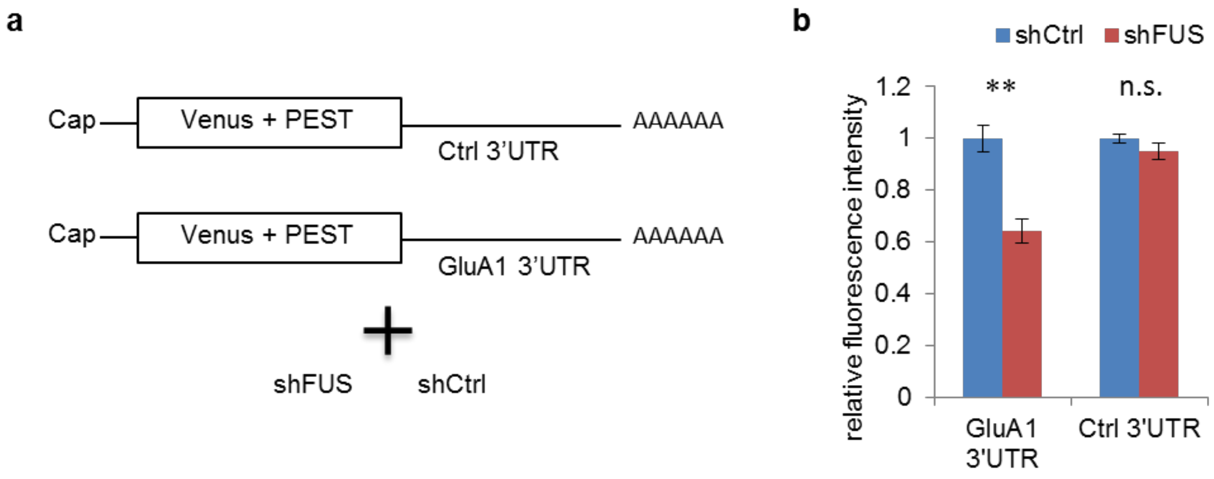

Supplementary Figure 6: 3' end of GluA1 mRNA 3'UTR is responsible for FUS-mediated reporter expression control. (**a**) Scheme of the reporter assay assessing the role of the GluA1 3' UTR sequences for the expression of Venus fluorescent protein fused with PEST sequence. 3'UTR of SCN1A whose expression in cultured neuron is not affected by FUS depletion was used as control. Each reporter construct was transfected into Neuro2A with shCtrl or shFUS lentivirus and assayed for fluorescence. (**b**) The level of the reporter gene expression was measured by fluorescence microscopy and analyzed by Image J software. (GluA1 3' UTR: $n = 3$ cultures for shCtrl, $n = 4$ cultures for shFUS, $t = 5.075$ **$P = 0.0039$; Ctrl 3' UTR: $n = 3$ cultures each, $t = 1.391$, $P = 0.27$, unpaired t-test)

Supplementary Figure 7

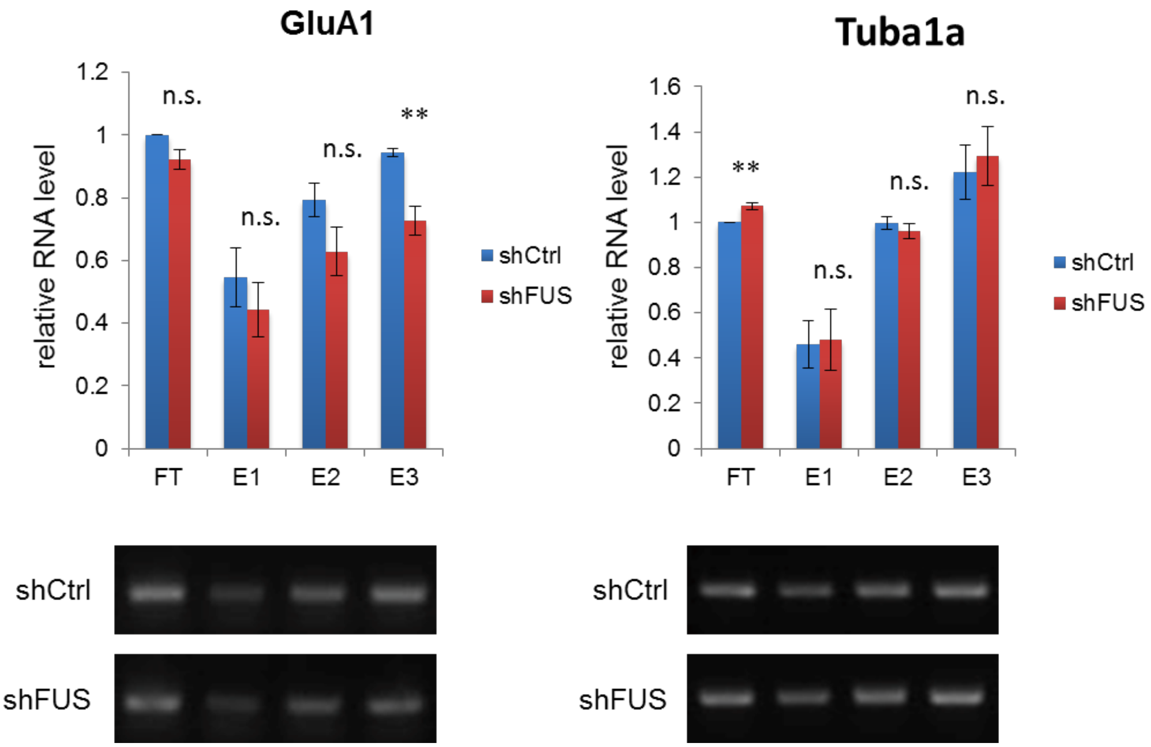

Supplementary Figure 7: GluA1 mRNA with long poly (A) tail is reduced upon FUS knockdown. Total RNA from control and FUS knockdown neurons were fractionated as described in Methods. The unbound RNA was washed with 0.5xSSC (FT) followed by serial elutions with buffers containing 0.2xSSC (E1), 0.075xSSC (E2), and distilled water (E3). GluA1 (**a**) and Tuba1a (**b**) mRNAs were detected by RT-PCR (n = 3). The band intensity was quantified by Image J software (GluA1; FT: t = 2.495, P = 0.0671, E1: t = 0.8077, P = 0.4645, E2: t = 1.773, P = 0.1510, E3: t = 4.620, P = 0.0099, Tuba1a; FT: t = 4.673, P = 0.0095, E1: t = 0.1257, P = 0.9061, E2: t = 0.8359, P = 0.4520, E3: t = 0.3956, P = 0.7126, unpaired t-test). E1, E2, and E3 fractions contains mRNAs with poly (A) tail of roughly less than 20 nt, 20-50 nt, and longer than 50 nt, respectively.

Supplementary Figure 8

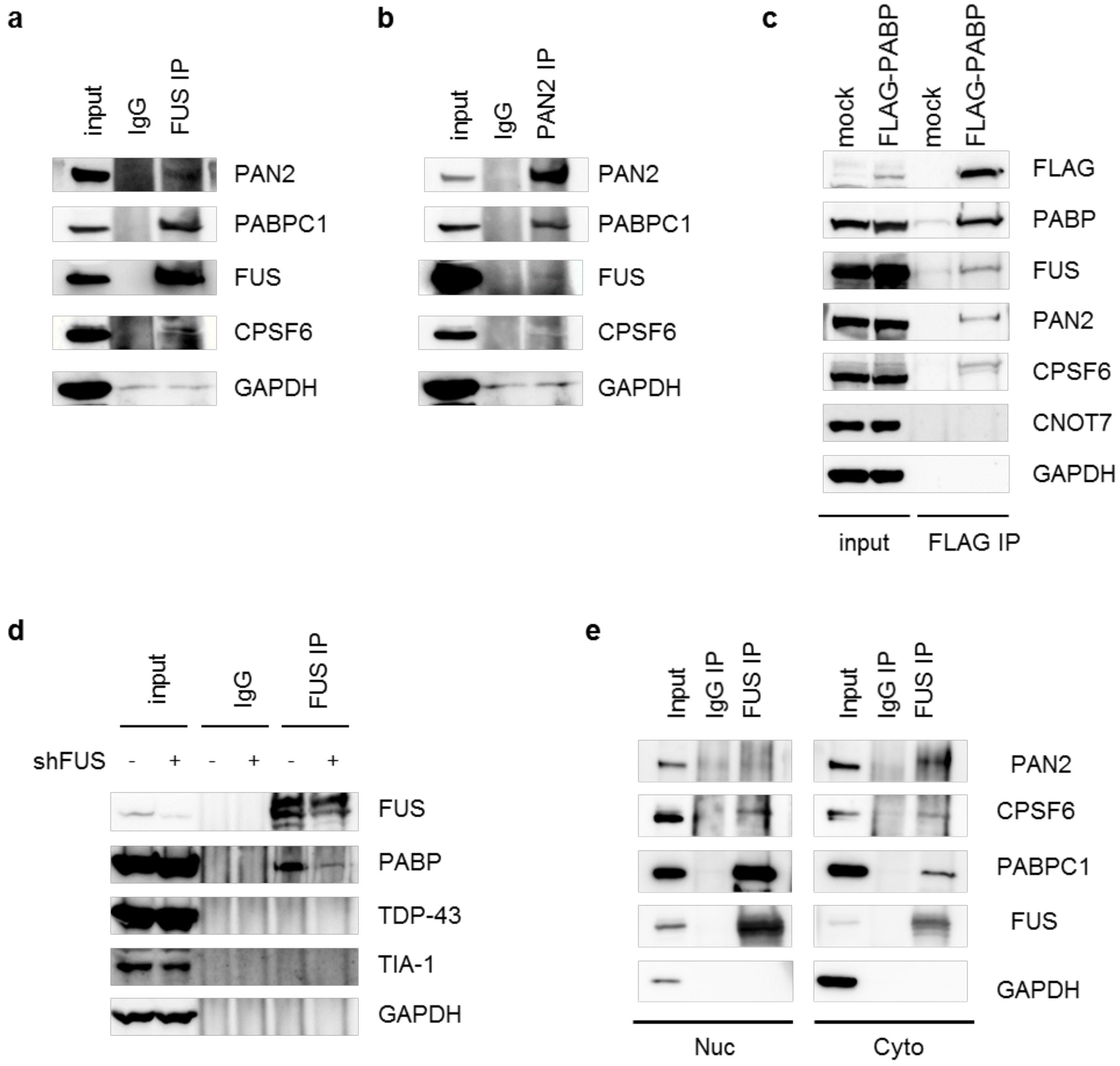

Supplementary Figure 8: FUS-containing protein complex in neuroblastoma cells and cultured neurons. (**a, b, c**) Co-immunoprecipitation of FUS (**a**), PAN2 (**b**), and FLAG-tagged PABPC1 (**c**) in neuroblastoma (Neuro2A) cells. (**d**) Co-immunoprecipitation of FUS in the control and FUS knockdown primary cortical neurons. (**e**) Co-immunoprecipitation of FUS in the nucleus and the cytoplasm of primary cortical neurons.

Supplementary Figure 9

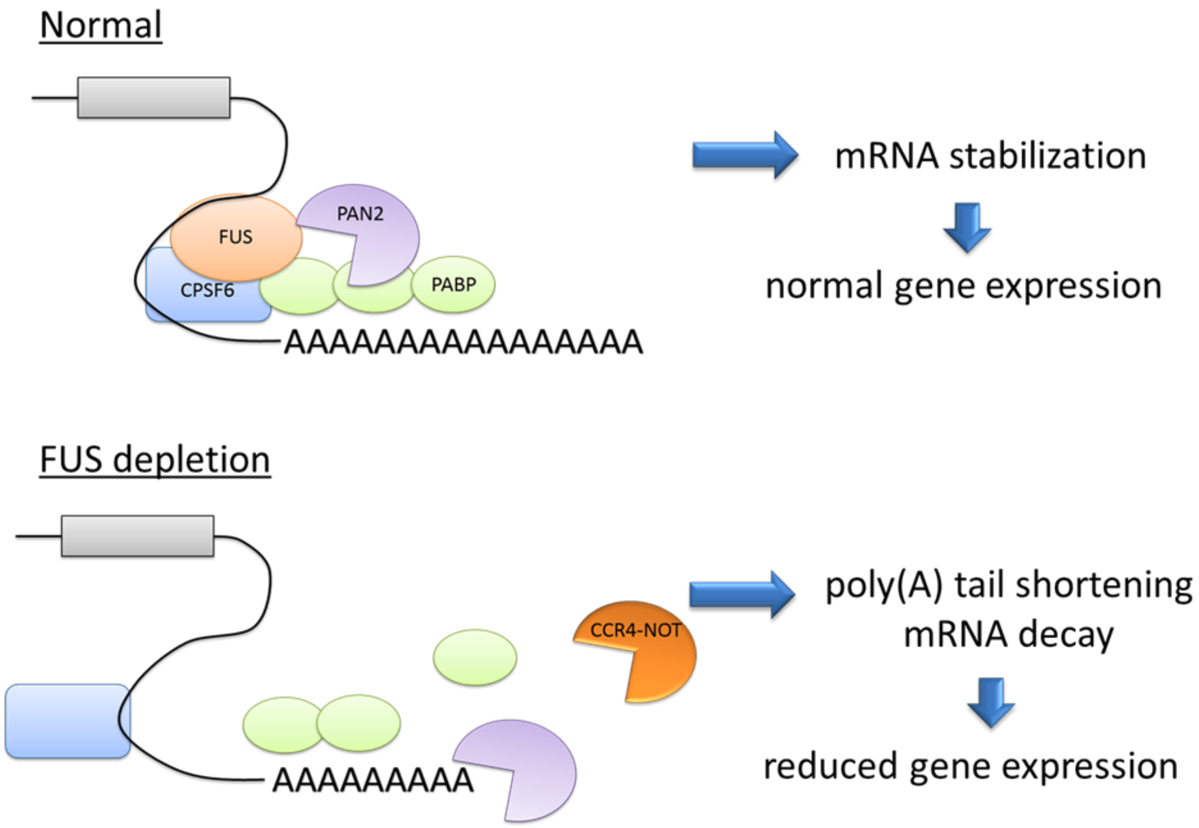

Supplementary Figure 9: Model for FUS-mediated poly (A) tail maintenance. FUS-containing 3' end processing machinery including FUS, PABP, PAN2, and CPSF6 stabilizes the poly (A) tail of the bound mRNA. In the absence of FUS, PAN2 and CPSF6 is release from the complex and PAN2 destabilizes the mRNA possibly by shortening the poly (A) tail.

Supplementary Figure 10

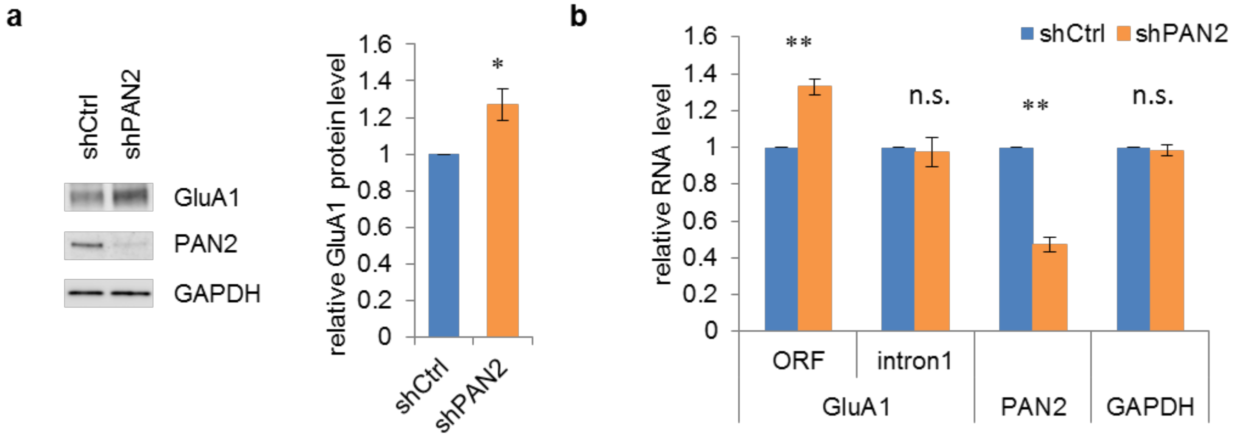

Supplementary Figure 10: PAN2 depletion up-regulates GluA1 by increasing its mature mRNA level. (**a**) GluA1 protein level was increased upon PAN2 knockdown. Primary cortical neurons, DIV 10, were infected by lentviruses expressiong shCtrl or shPAN2 and the lysates were prepared 10 days later and analyzed by western blot (n = 3 each, t = 4.417 *P = 0.048, paired t-test). (**b**) Mature, but not primary, GluA1 transcript was increased upon PAN2 depletion. After the PAN2 knockdown, RNA was extracted and analyzed by qPCR using primer sets in Fig. 2a (n = 4 each, ORF: t = 7.802, **P = 0.0044; intron1: t = 0.3347, P = 0.76; PAN2: t = 12.84, **P = 0.0010; GAPDH: t = 0.5608, P = 0.61, paired t-test)

Supplementary Figure 11

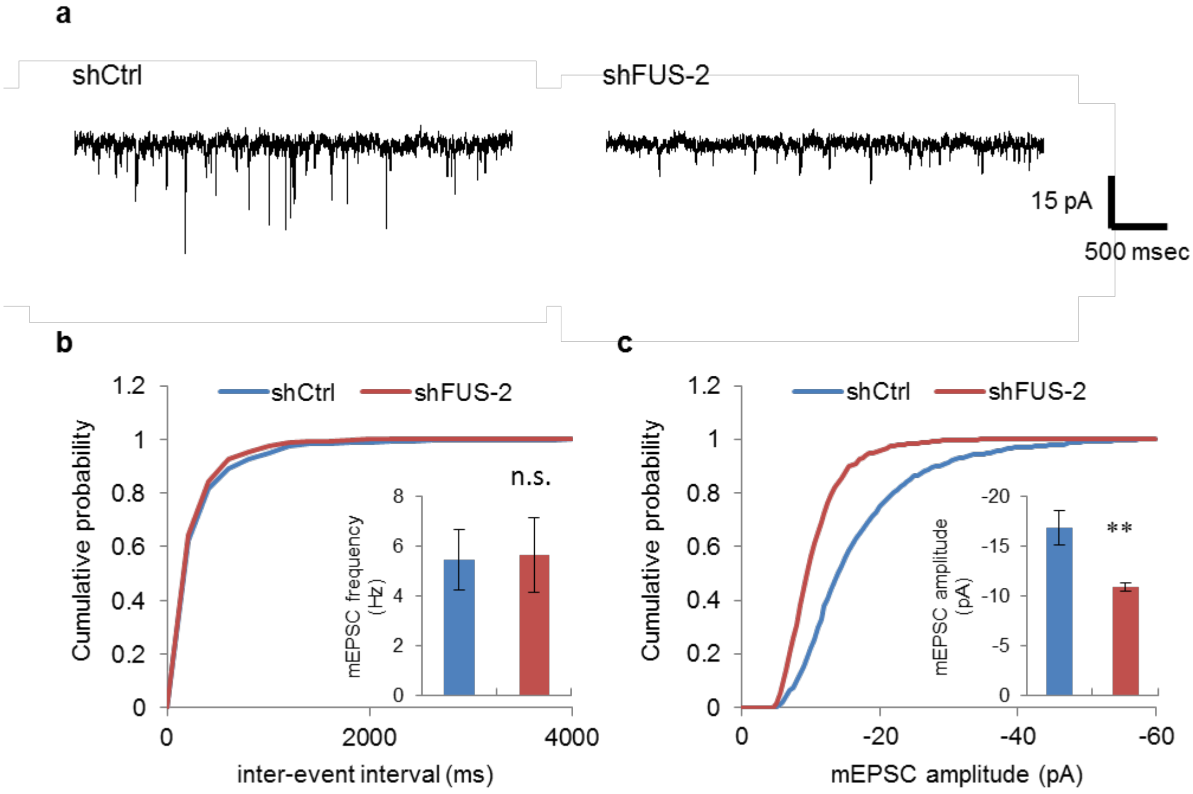

Supplementary Figure 11: Second shRNA against FUS reduces mEPSC amplitude but not frequency. (**a**) Representative mEPSC sample traces from the shCtrl (*n* = 7) and shFUS-2 (*n* = 7) cultured cortical neurons. 1-2 cells per separate culture were used for experiments. Calibration: 15pA, 500ms. (**b, c**) Cumulative distributions of mEPSC frequency (**b**) and amplitude (**c**) from the shCtrl and shFUS-2 cortical neurons. The insets show mean ± SEM for mEPSC frequency (t = 0.08484, P = 0.93, unpaired *t*-test) (**b**) and amplitude (t = 3.353 **P = 0.0057, unpaired *t*-test) (**c**).

Supplementary Figure 12

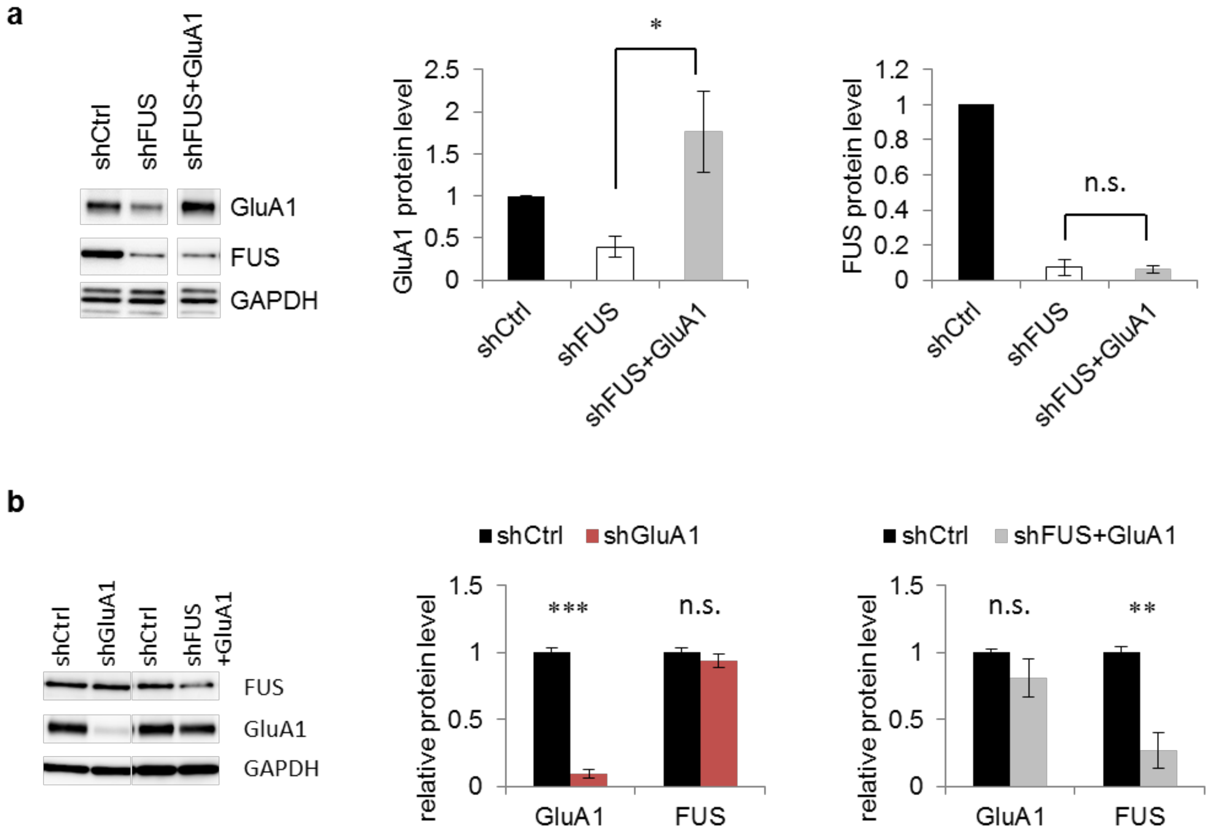

Supplementary Figure 12: Exogenous GluA expression in cultured neurons and the hippocampus. (**a**)Western blot of the lysates from control, FUS knockdown, and FUS knockdown with exogenous GluA1 expression hippocampal neurons showed that exogenous GluA1 expression recovered total GluA1 level in FUS knockdown (n = 3 each, $F(2, 6) = 5.665$, *P = 0.042, one-way ANOVA; shFUS vs shFUS+GluA1: *P = 0.035, Tukey's test post hoc), while FUS level was not affected (n = 3 each, $F(2, 6) = 375.3$, *P < 0.0001, one-way ANOVA; shFUS vs shFUS+GluA1: P = 0.97, Tukey's test post hoc). (**b**) Western blot of the microdissected hippocampus from the brain slices of the GluA1 knockdown (n = 3 each, GluA1: t = 19.72, ***P < 0.0001; FUS: t = 1.070, P = 0.35, unpaired t-test) and the FUS knockdown with

GluA1 expression mice (*n* = 3 each, GluA1: *t* = 1.332, *P* = 0.25; FUS: *t* = 5.169, $^{**}P = 0.0067$, unpaired *t*-test).

Supplementary Figure 13

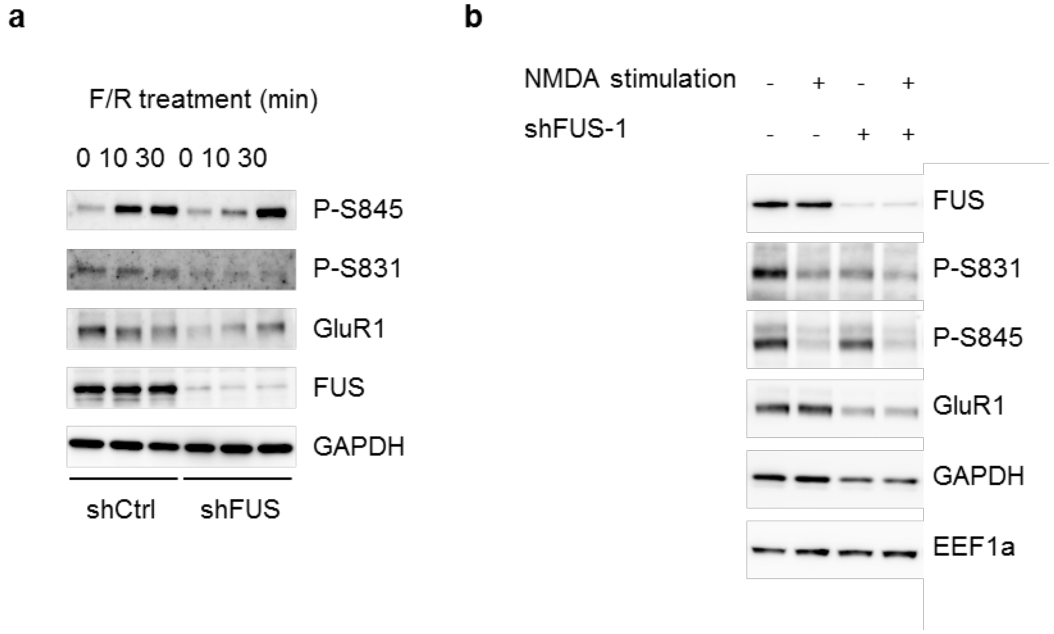

Supplementary Figure 13: FUS depletion retains activity-induced GluA1 phosphorylation at serine 845. (**a**) Cultured cortical neurons transduced by shCtrl or shFUS were stimulated by forskolin/rolipram and GluR1 phosphorylation was analyzed by western blotting using phospho-specific antibodies at serine 845 and 831. (**b**) Cultured neurons were stimulated with 50 μM NMDA and analyzed as described in (a).

Supplementary Figure 14

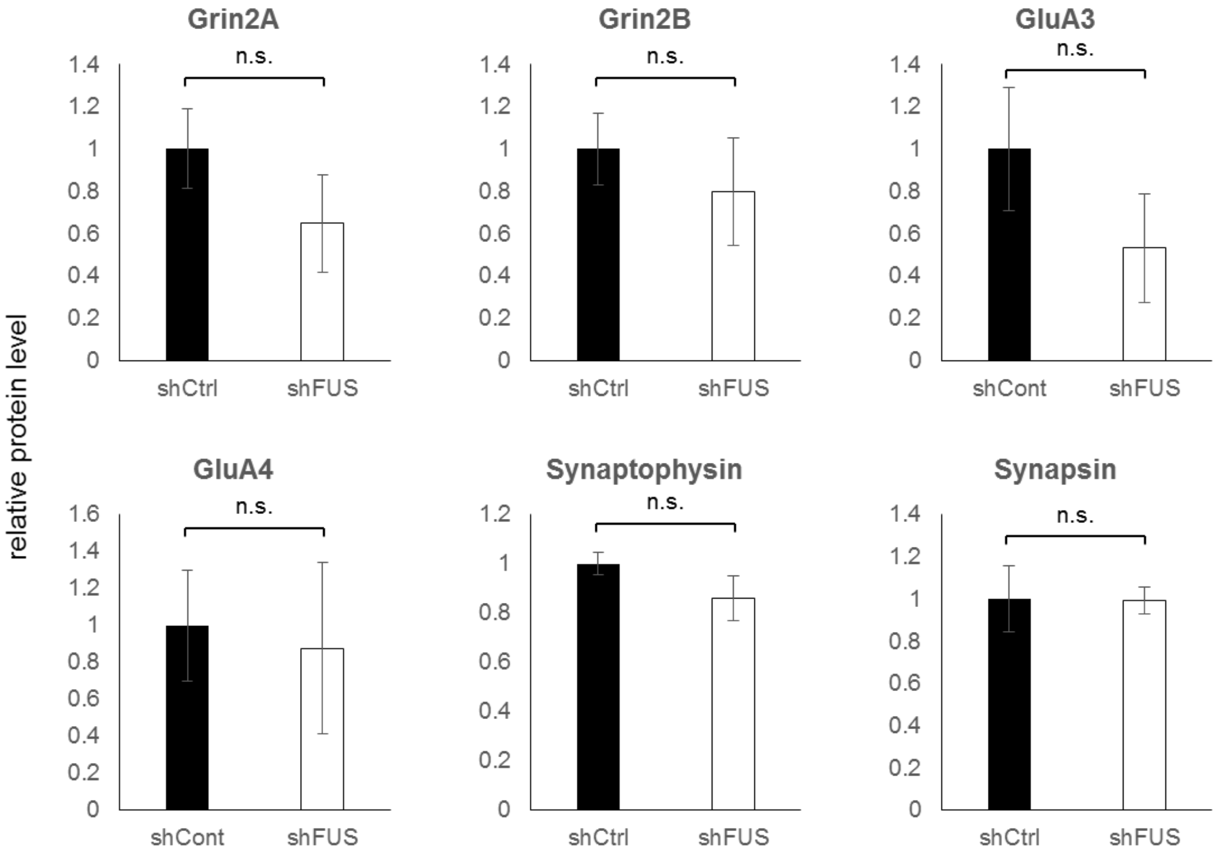

Supplementary Figure 14: The lysates were prepared from the GFP-positive hippocampal slices and western blotted with the indicated antibodies. Protein levels were quantified in four control and four FUS knockdown mice (Grin2A: t = 1.180, P = 0.2825; Grin2B: t = 4.868e-8, P > 0.999; GluA3: t = 1.204, P = 0.2740; GluA4: t = 0.2244, P = 0.8299; Synaptophysin: t = 1.369, P = 0.2200; Synapsin: t = 0.04746, P = 0.9637, unpaired t-test).

Supplementary Figure 15

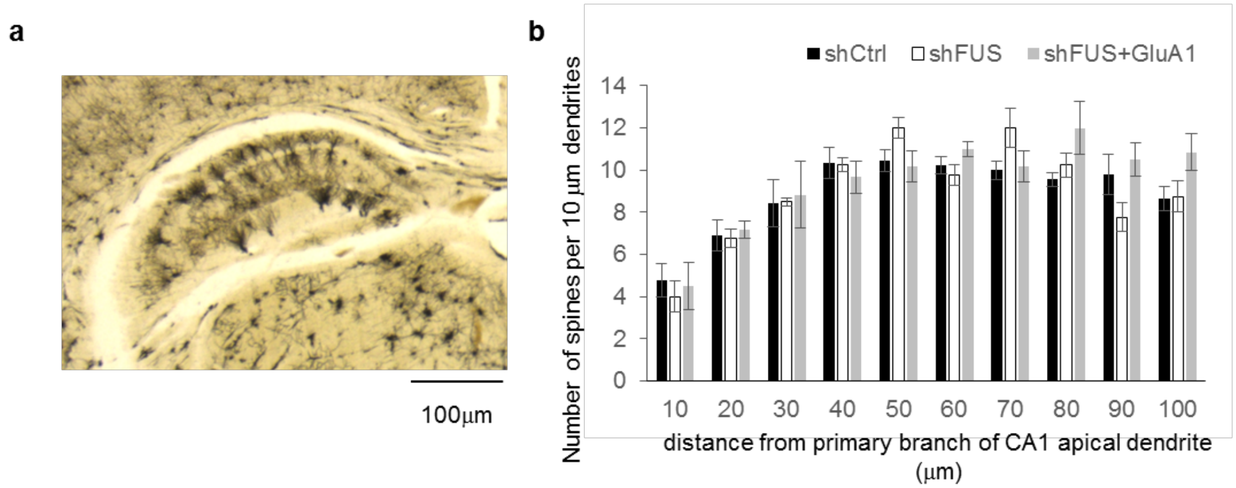

Supplementary Figure 15: Gogi-Cox staining of AAV-injected mice. (**a**) Representative low-power field image of Golgi-Cox staining of AAV-injected mouse hippocampus. (**b**) Segmental analysis of dendritic spine density along a primary dendritic branch of CA1 pyramidal neurons. Total number of spines along the dendritic branch is shown for 10 μm segments starting from the origin of the branch to 100 μm from the origin. No significant difference was detected (*n* = 9 (shCtrl), 9 (shFUS), and 6 (shFUS+GluA1), one-way ANOVA).

Supplementary Figure 16

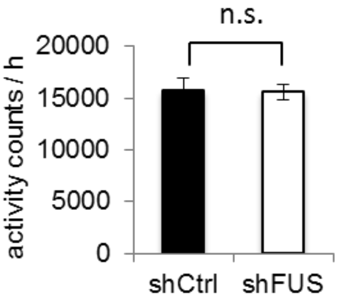

Supplementary Figure 16: Homecage activity of the AAV-injected mice. To assess basal locomotor activity, homecage activity was measured in shCtrl and shFUS injected mice. No statistical difference was observed. (n = 12 each, t = 0.08957, P = 0.93, unpaired t-test)

Supplementary Figure 17

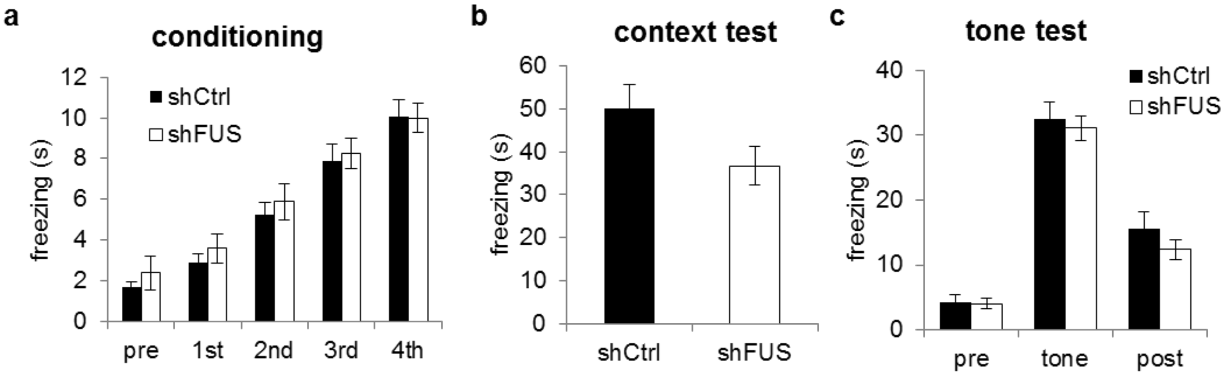

Supplementary Figure 17: Fear conditioning test for FUS knockdown group. (**a**) There was no difference between control (*n* = 22) and FUS knockdown (*n* = 18) during conditioning phase (Mann-Whitney's U test). (**b**, **c**) Neither contextual (**b**) nor cued (**c**) fear memory was significantly affected by FUS knockdown (unpaired *t*-test and Mann-Whitney's U test, respectively).

Supplementary Figure 18

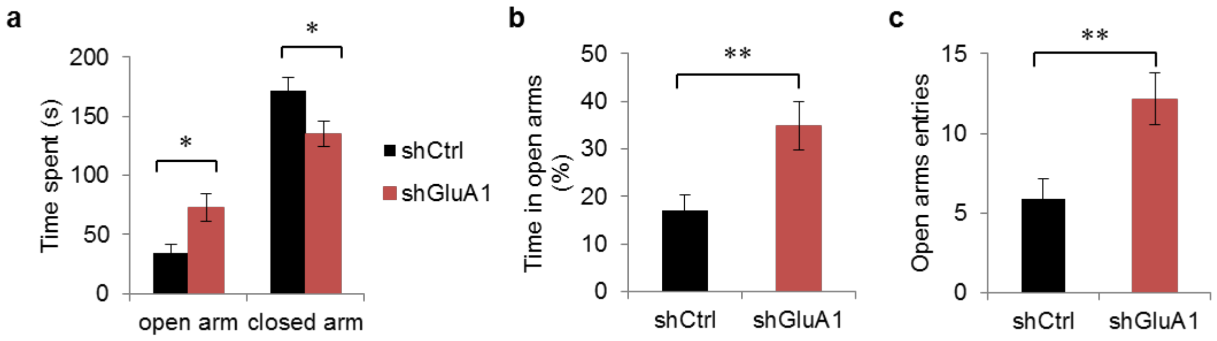

Supplementary Figure 18: Elevated plus maze test for GluA1 knockdown group. Elevated plus maze test for shCtrl (*n* = 11) and shGluR1 (*n* = 13) groups was performed as described in Fig. 8a, b and c. GluA1 knockdown increased open arm duration (**a, b**) and entry (**c**) (open arm duration: *t* = 2.743, *P* = 0.012; closed arm duration: *t* = 2.305, *P* = 0.031; open arm ratio: *t* = 2.825, *P* = 0.0099; open arm entries: *t* = 2.977, *P* = 0.0069, unpaired *t*-test).

Supplementary Figure 19

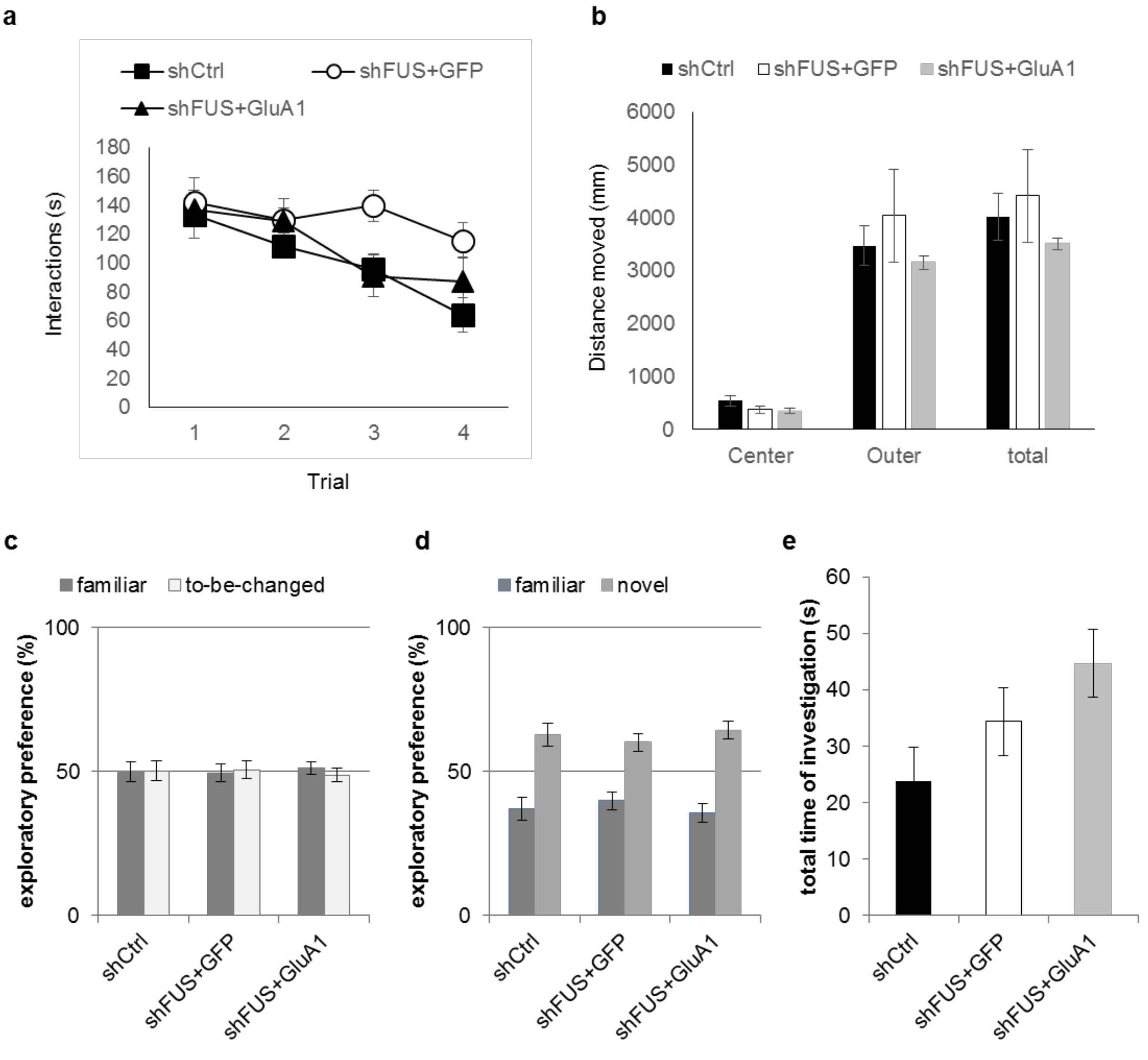

Supplementary Figure 19: Behavioral assay of FUS knockdown mice with GluA1 expression. (**a**) Resident-intruder test measuring the investigation time of the test mouse of the AAV-shCtrl-, AAV-shFUS/GFP-, or AAV-shFUS/GluA1-injected group on an intruder mouse in four consecutive sessions (session 1: $F(2, 22) = 0.08766$, $P = 0.9164$, session 2: $F(2, 22) = 0.8145$, $P = 0.4558$, session 3: $F(2, 21) = 5.972$, $^{**}P = 0.0088$, session 4: $F(2, 22) = 4.214$, $^{*}P = 0.0282$, one-way ANOVA). (**b**) Distance moved in the indicated compartment of an open field

during the 5 min test session (total: $F(2, 22) = 0.6249$, $P = 0.5446$; center: $F(2, 22) = 1.735$, $P = 0.1996$; outer: $F(2, 22) = 0.6439$, $P = 0.5349$; one-way ANOVA). (**c-e**) Novel object recognition test. (**c**) In the training session, all groups equally investigated two different objects placed in the test field. ($P > 0.05$, unpaired *t*-test) (**d**) During the retention session where one object was replaced with a novel object, all groups spent significantly more time investigating the novel object (shCtrl: $n = 9$, $t = 4.539$, ***$P = 0.0003$; shFUS+GFP: $n = 8$, $t = 4.551$, ***$P = 0.0005$; shFUS+GluA1: $n = 8$, $t = 6.403$, ***$P < 0.0001$, unpaired *t*-test). (**e**) Total investigation time on the two objects presented in training session ($F(2,22) = 1.954$, $P = 0.1655$, one-way ANOVA).

Supplementary Figure 20

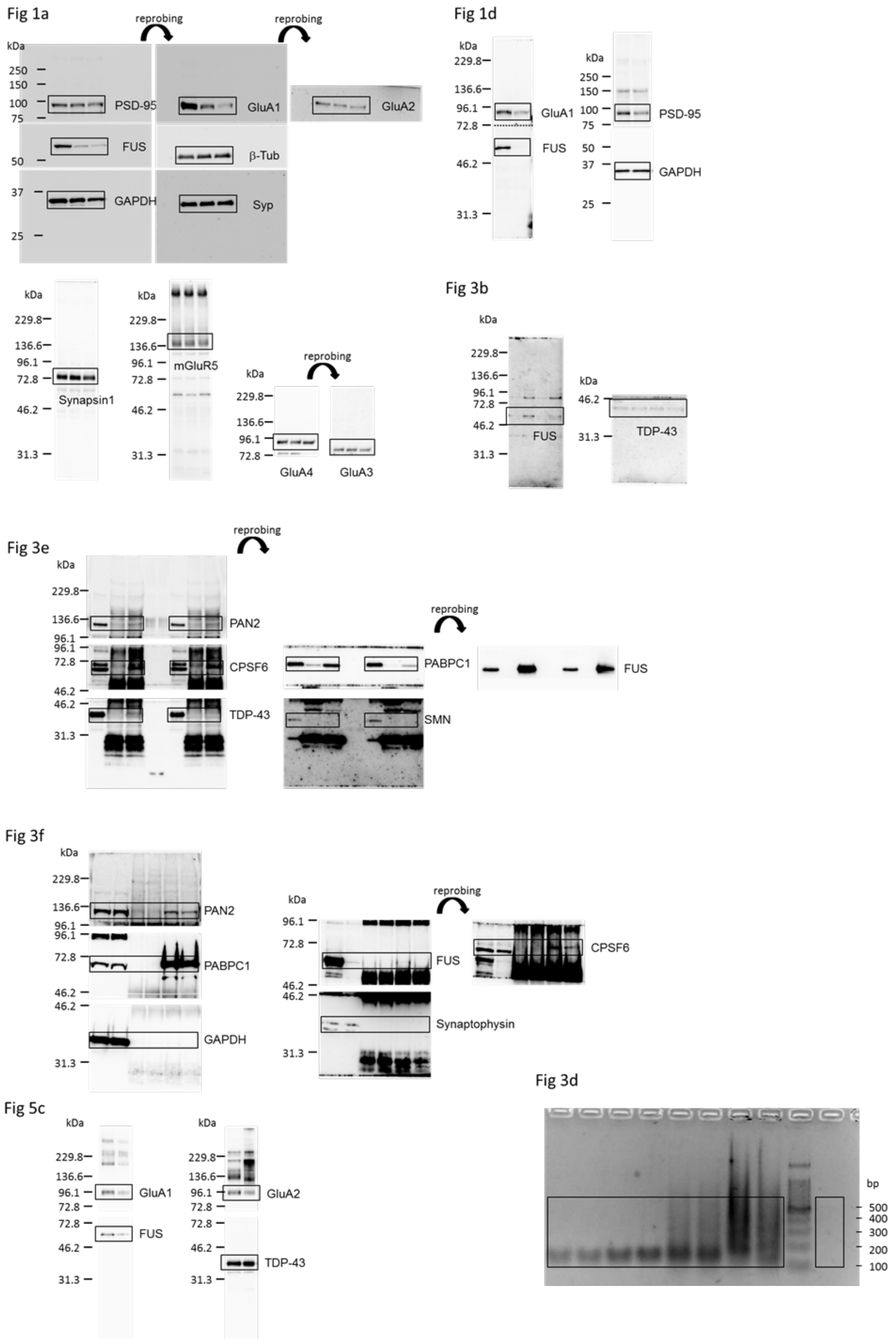

Supplementary Figure 20: Original blots and gel in Figures 1a, d, 3b, d, e, f, and 5c.

Supplemental Table 1

List of FUS-interacting proteins identified in NSC-34 cells that have been implicated in mRNA processing, stability, transport , and translation in cytoplasm. Those that are involved in splicing were excluded. Proteins examined in Figure 3 were highlighted in yellow.

Accession #	Description
P97351	40S ribosomal protein S3a OS=Mus musculus GN=Rps3a PE=1 SV=3 - [RS3A_MOUSE]
P29341	Polyadenylate-binding protein 1 OS=Mus musculus GN=Pabpc1 PE=1 SV=1 - [PABP1_MOUSE]
P56959	RNA-binding protein FUS OS=Mus musculus GN=Fus PE=2 SV=1 - [FUS_MOUSE]
P97801	Survival motor neuron protein OS=Mus musculus GN=Smn1 PE=1 SV=1 - [SMN_MOUSE]
P35980	60S ribosomal protein L18 OS=Mus musculus GN=Rpl18 PE=2 SV=3 - [RL18_MOUSE]
P63276	40S ribosomal protein S17 OS=Mus musculus GN=Rps17 PE=1 SV=2 - [RS17_MOUSE]
P14115	60S ribosomal protein L27a OS=Mus musculus GN=Rpl27a PE=2 SV=5 - [RL27A_MOUSE]
P47911	60S ribosomal protein L6 OS=Mus musculus GN=Rpl6 PE=1 SV=3 - [RL6_MOUSE]
P97461	40S ribosomal protein S5 OS=Mus musculus GN=Rps5 PE=2 SV=3 - [RS5_MOUSE]
P86048	60S ribosomal protein L10-like OS=Mus musculus GN=Rpl10l PE=2 SV=1 - [RL10L_MOUSE]
P14148	60S ribosomal protein L7 OS=Mus musculus GN=Rpl7 PE=1 SV=2 - [RL7_MOUSE]
Q61701	ELAV-like protein 4 OS=Mus musculus GN=Elavl4 PE=1 SV=1 - [ELAV4_MOUSE]
Q61584	Fragile X mental retardation syndrome-related protein 1 OS=Mus musculus GN=Fxr1 PE=1 SV=2 - [FXR1_MOUSE]
Q60899	ELAV-like protein 2 OS=Mus musculus GN=Elavl2 PE=2 SV=1 - [ELAV2_MOUSE]
Q60900	ELAV-like protein 3 OS=Mus musculus GN=Elavl3 PE=1 SV=1 - [ELAV3_MOUSE]
P12970	60S ribosomal protein L7a OS=Mus musculus GN=Rpl7a PE=2 SV=2 - [RL7A_MOUSE]
P32067	Lupus La protein homolog OS=Mus musculus GN=Ssb PE=2 SV=1 - [LA_MOUSE]
Q921F2	TAR DNA-binding protein 43 OS=Mus musculus GN=Tardbp PE=1 SV=1 - [TADBP_MOUSE]
Q9D0I8	mRNA turnover protein 4 homolog OS=Mus musculus GN=Mrto4 PE=2 SV=1 - [MRT4_MOUSE]
Q61990	Poly(rC)-binding protein 2 OS=Mus musculus GN=Pcbp2 PE=1 SV=1 - [PCBP2_MOUSE]
P35979	60S ribosomal protein L12 OS=Mus musculus GN=Rpl12 PE=1 SV=2 - [RL12_MOUSE]
P57722	Poly(rC)-binding protein 3 OS=Mus musculus GN=Pcbp3 PE=2 SV=3 - [PCBP3_MOUSE]
Q6ZQ08	CCR4-NOT transcription complex subunit 1 OS=Mus musculus GN=Cnot1 PE=1 SV=2 - [CNOT1_MOUSE]
Q6NVF9	Cleavage and polyadenylation specificity factor subunit 6 OS=Mus musculus GN=Cpsf6 PE=1 SV=1 - [CPSF6_MOUSE]
P27659	60S ribosomal protein L3 OS=Mus musculus GN=Rpl3 PE=2 SV=2 - [RL3_MOUSE]
Q6ZQ58	La-related protein 1 OS=Mus musculus GN=Larp1 PE=1 SV=2 - [LARP1_MOUSE]
P51410	60S ribosomal protein L9 OS=Mus musculus GN=Rpl9 PE=2 SV=2 - [RL9_MOUSE]
P35922	Fragile X mental retardation protein 1 homolog OS=Mus musculus GN=Fmr1 PE=1 SV=1 - [FMR1_MOUSE]
Q9D8E6	60S ribosomal protein L4 OS=Mus musculus GN=Rpl4 PE=1 SV=3 - [RL4_MOUSE]
P70372	ELAV-like protein 1 OS=Mus musculus GN=Elavl1 PE=1 SV=1 - [ELAV1_MOUSE]
Q9CPN8	Insulin-like growth factor 2 mRNA-binding protein 3 OS=Mus musculus GN=Igf2bp3 PE=1 SV=1 - [IF2B3_MOUSE]
Q6NZJ6	Eukaryotic translation initiation factor 4 gamma 1 OS=Mus musculus GN=Eif4g1 PE=1 SV=1 - [IF4G1_MOUSE]
Q9WVR4	Fragile X mental retardation syndrome-related protein 2 OS=Mus musculus GN=Fxr2 PE=1 SV=1 - [FXR2_MOUSE]
P58252	Elongation factor 2 OS=Mus musculus GN=Eef2 PE=1 SV=2 - [EF2_MOUSE]
Q8BGF7	PAB-dependent poly(A)-specific ribonuclease subunit 2 OS=Mus musculus GN=Pan2 PE=2 SV=1 - [PAN2_MOUSE]
Q7TMB8	Cytoplasmic FMR1-interacting protein 1 OS=Mus musculus GN=Cyfip1 PE=1 SV=1 - [CYFP1_MOUSE]
Q5SQX6	Cytoplasmic FMR1-interacting protein 2 OS=Mus musculus GN=Cyfip2 PE=1 SV=2 - [CYFP2_MOUSE]

Supplemental Table 2

Summary of behavioral phenotypes in this study and those previously reported for GluA1 knockout mice(*). Phonotypes that were commonly affected in FUS KD and GluA1 KO and were resuced by GluA1 expression were highlighted in pink. Phenotype that were affected in FUS KD and GluA1 KO, but not rescued by GluA1 were highlighted in blue.

Behavioral phenotype	FUS KD	GluA1 KO*	GluA1 KD	Rescued GluA1 KD
Enhanced locomotor activity in OFT#	Yes	Yes	N.T.	Yes‡
Enhanced investigation of novel object in NOR#	Yes	Yes	N.T.	No
Impaired novel object recognition in standard NOR#	No	No	N.T.	-
Enhanced open arm duration of EPM#	Yes	Yes	Yes	Yes
Increased entries into open arm of EPM#	Yes	Yes	Yes	Yes
Exaggerated social interaction	Yes	Yes	N.T.	Yes‡
aggression	No	reduced	N.T.	-
Impaired fear conditioning	No	Yes	N.T.	-
Spatial working memory deficit	N.T.	Yes	N.T.	N.T.

OFT, open field test; NOR, novel object recognition test; EPM, elevated plus maze test
‡ $P > 0.05$

Supplemental Table 3

Antibodies used in this study

antibody	company	catalog number	dilution
Actin	Sigma	A2066	1:1000 (WB)
CNOT7	Abcam	ab57095	1:500 (WB)
CPSF6	Bethyl Laboratories	A301-356A-1	1:500 (WB)
FUS	Bethyl Laboratories	A300-293	1:3000 (WB), 1:5000 (IF)
FUS	Santa Cruz	sc-47711	1:1000 (WB)
GAPDH	MBL	M171-3	1:2000 (WB)
GluA1	Millipore	AB1504	1:1000 (WB), 1:1000 (IF), 1:50 (IHC)
GluA1	Santa Cruz	sc-13152	1:500 (WB)
GluA2	Millipore	MAB397	1:2000 (WB)
GluA3	Millipore	MAB5416	1:500 (WB)
GluA4	Millipore	AB1508	1:500 (WB)
Grin2A	Millipore	07-632	1:500 (WB)
Grin2B	Millipore	06-600	1:500 (WB)
MAP2	Abcam	ab5392	1:10000 (IF)
PABPC1	Abcam	ab21060	1:500 (WB)
PAN2	Proteintech	16427-1-AP	1:500 (WB)
PSD-95	BD Transduction	610496	1:250 (WB)
Pum2	Bethyl Laboratories	A300-202A	1:200 (WB)
SMN	Millipore	05-1532	1:200 (WB)
Synaptophysin	Abcam	ab8049	1:1000 (WB)
Synapsin	Abcam	ab8	1:1000 (WB)
TDP-43	Proteintech	10782-2-AP	1:3000 (WB)
TIA1	Santa Cruz	sc-1751	1:100 (WB)
beta-Tubulin	Abcam	TU-20	1:1000 (WB)

Supplemental Table 4

qPCR primer sequences

name	sequences	target
GluA1-ORF-F	ATGCTGACCTCCTTCTGTGG	GluA1 ORF
GluA1-ORF-R	TGCCACTTGTAATGGTCGAT	GluA1 ORF
GluA1-RT-F	GAGGAGGGCATGATCAGAGT	GluA1 flip and flop
GluA1-flip-R	ACTTCCGGAGTCCTTGCTTC	GluA1 flip
GluA1-flop-R	GGAGTCACCTCCCCCGCTGC	GluA1 flop
GluA1-3'UTR-F	CTTTGGTACCTGGGCCTTTT	GluA1 3'UTR
GluA1-3'UTR-R	AGAAAAACCCTGGCTATTTGAA	GluA1 3'UTR
GluA1-intron1-F	ATTCCTGGCTTCCATCCTCT	GluA1 intron 1
GluA1-intron1-R	CTGTTCCCATGCTGATGTTG	GluA1 intron 1
GluA1-intron2-F	TAAGCCTGTGCTTTCGGAGT	GluA1 intron 2
GluA1-intron2-R	TAGGCGAATGAACTTGGACC	GluA1 intron 2
Tap1-F	CTTCAGTTCACCCAGGCTGT	Tap1
Tap1-R	CAAGGAGTCCGGTCCAAGTA	Tap1
GAPDH-RT-F	GGTGAAGGTCGGTGTGAACG	GAPDH
GAPDH-RT-R	CTCGCTCCTGGAAGATGGTG	GAPDH
Tuba1a-RT-F	GCTTCTTGGTTTTCCACAGC	Tuba1a
Tuba1a-RT-R	TGGAATTGTAGGGCTCAACC	Tuba1a
B-Actin-F	GCAAGTGCTTCTAGGCGGAC	B-Actin
B-Actin-R	AAGAAAGGGTGTAAAACGCAGC	B-Actin
FUS-F	GGCTACTCCCAACAGAGCAG	FUS
FUS-R	GCTGTTTTGGGTCTGTCCAT	FUS
